# Nervous system-wide analysis of Hox regulation of terminal neuronal fate specification in *Caenorhabditis elegans*

**DOI:** 10.1371/journal.pgen.1010092

**Published:** 2022-02-28

**Authors:** Chaogu Zheng, Ho Ming Terence Lee, Kenneth Pham

**Affiliations:** 1 School of Biological Sciences, The University of Hong Kong, Hong Kong SAR, China; 2 Department of Biological Sciences, Columbia University, New York, New York, United States of America; University of California San Diego, UNITED STATES

## Abstract

Hox genes encode evolutionarily conserved transcription factors that specify regional identities along the anterior-posterior (A-P) axis. Although some Hox genes are known to regulate the differentiation of certain neurons, to what extent Hox genes are involved in the terminal specification of the entire nervous system is unclear. Here, we systematically mapped the expression of all six Hox genes in *C*. *elegans* nervous system and found Hox expression in 97 (32%) of the 302 neurons in adult hermaphrodites. Our results are generally consistent with previous high-throughput expression mapping and single-cell transcriptomic studies. Detailed analysis of the fate markers for these neurons revealed that Hox genes regulate the differentiation of 29 (25%) of the 118 classes of *C*. *elegans* neurons. Hox genes not only regulate the specification of terminal neuronal fates through multiple mechanisms but also control subtype diversification along the A-P axis. The widespread involvement of Hox genes in neuronal differentiation indicates their roles in establishing complex nervous systems.

## Author summary

The nervous system contains an extraordinary array of neuron types. How this neuronal diversity arises during development and what genes regulate the differentiation of each neuron type are among the major questions of neurobiology. Hox genes are a set of transcription factors highly conserved in the animal kingdom and are involved in setting up the body plan in the embryos. Hox genes are known to regulate the differentiation of some neurons, but their contribution to the overall development of a nervous system is unclear. In this study, we analyzed the activity of the Hox genes in the differentiation of the 302 neurons of the *C*. *elegans* nervous system in its entirety. We found that the six Hox genes are expressed in 32% of all neurons and five Hox genes regulate the differentiation of 25% of all neuron types through multiple mechanisms and act at various stages of cellular development. Thus, our results suggest that a small number of Hox genes could control the development of a significant portion of the nervous system. Given the conserved functions of Hox genes across species, we suspect that the increasing number of Hox genes may have allowed increased complexity in the nervous system in animal evolution.

## Introduction

Hox genes are a group of evolutionarily conserved homeodomain transcription factors that specify regional identities along the anterior-posterior (A-P) axis during development [1]. Hox genes have highly ordered expression along the A-P axis, which makes them the central organizers of many developmental processes, including embryonic body planning, axial identity patterning, and early neural patterning [2]. For example, the *Drosophila* Hox gene, *Antp*, specifies the identity of the second thoracic segment (T2) and promotes leg formation; the loss of *Antp* transforms T2 to a more anterior identity and converts the second leg to an antenna, and ectopic expression of *Antp* in the head converts the antenna to the second leg [3,4]. Such posterior dominance among the Hox genes is an important mechanism for the specification of regional identities.

Hox genes also play important roles in regulating neuronal differentiation. In *Drosophila* embryonic ventral nerve cord, Hox genes control the specification of peptidergic neurons [5]. In *C*. *elegans*, we previously found that Hox genes play dual functions in both promoting cell fate specification and subtype diversification along the A-P axis in the touch receptor neurons [6,7]. Similarly, Hox genes also regulate the specification of HSN neurons [8] and control subtype identities along the A-P axis in the cholinergic motor neurons [9]. In vertebrates, Hox genes not only instruct neural patterning during neurogenesis but also regulate the subtype specification of diverse population of motor neurons in the hindbrain and the spinal cord by controlling axon trajectories and circuit connectivity [10]. These findings suggest that the function of Hox genes in terminal neuronal fate determination and subtype specification is highly conserved across animal species.

Despite the above piecemeal studies of Hox activities in particular neuron types, efforts to systematically understand Hox regulation of neuronal specification in the entire nervous system are still lacking. How many neurons are regulated by Hox genes during fate specification? Do the Hox genes regulate these neurons through similar mechanisms? In this study, we systematically mapped the expression of the six Hox genes in the *C*. *elegans* nervous system at the single-cell resolution. We found that 97 (32%) of the 302 neurons, or 29 (25%) of the 118 neuron types, in hermaphrodite adults expressed at least one Hox gene. Among the 29 neuron types, we found direct evidence for Hox regulation of neuronal fate specification or subtype diversification in 22 neuron types, suggesting a significant contribution of the Hox genes in the generation of neuronal diversity in the nervous system. Moreover, we found that Hox genes also show varying mechanisms of regulation of cell fate in different neuron types and in different subtypes of the same neuron type. In summary, our study provides a system-level understanding for the involvement of Hox genes in specifying terminal neuronal fates.

## Results

### Mapping the expression of Hox genes in the nervous system

*C*. *elegans* genome contains six Hox genes, *ceh-13/Hox1*, *lin-39/Hox4-5*, *mab-5/Hox6*, *egl-5/Hox7-8*, *php-3/Hox10*, and *nob-1/Hox12*. To map their expression among the 302 terminally differentiated neurons in hermaphrodite adults, we used either fosmid-based translational reporters or GFP knock-in at their endogenous loci and crossed these reporters with the neurotransmitter identity markers and specific cell fate markers for cell identification (see Materials and Methods).

For *ceh-13*, we found that the fosmid-based reporter *wgIs756[ceh-13*::*EGFP]* was only expressed in the embryos and no significant GFP signal was detected in larvae or adult animals (S1A Fig). However, Tihanyi *et al*. [11] reported that a transcriptional reporter *ceh-13p*::*GFP*, which contains 8.1 kb sequence upstream of the start codon, was expressed in larval stages and in many cell lineages during development. To investigate this discrepancy, we generated a GFP knock-in at the *ceh-13* locus through CRISPR/Cas9-mediated gene editing and found that the expression of endogenous *ceh-13*::*GFP* was only observed in the early embryos, similar to *wgIs756[ceh-13*::*EGFP]* (S1B Fig). Although we occasionally observed very weak *ceh-13*::*GFP* expression in some presumably ventral nerve cord motor neurons in adults (S1C Fig), we were not able to consistently identify the neurons that showed the weak GFP signal. So, we concluded that the expression of *ceh-13* is mostly restricted to the embryonic stages, and its expression in terminally differentiated cells is rather weak and may be below our detection limit (see below).

**Fig 1:**
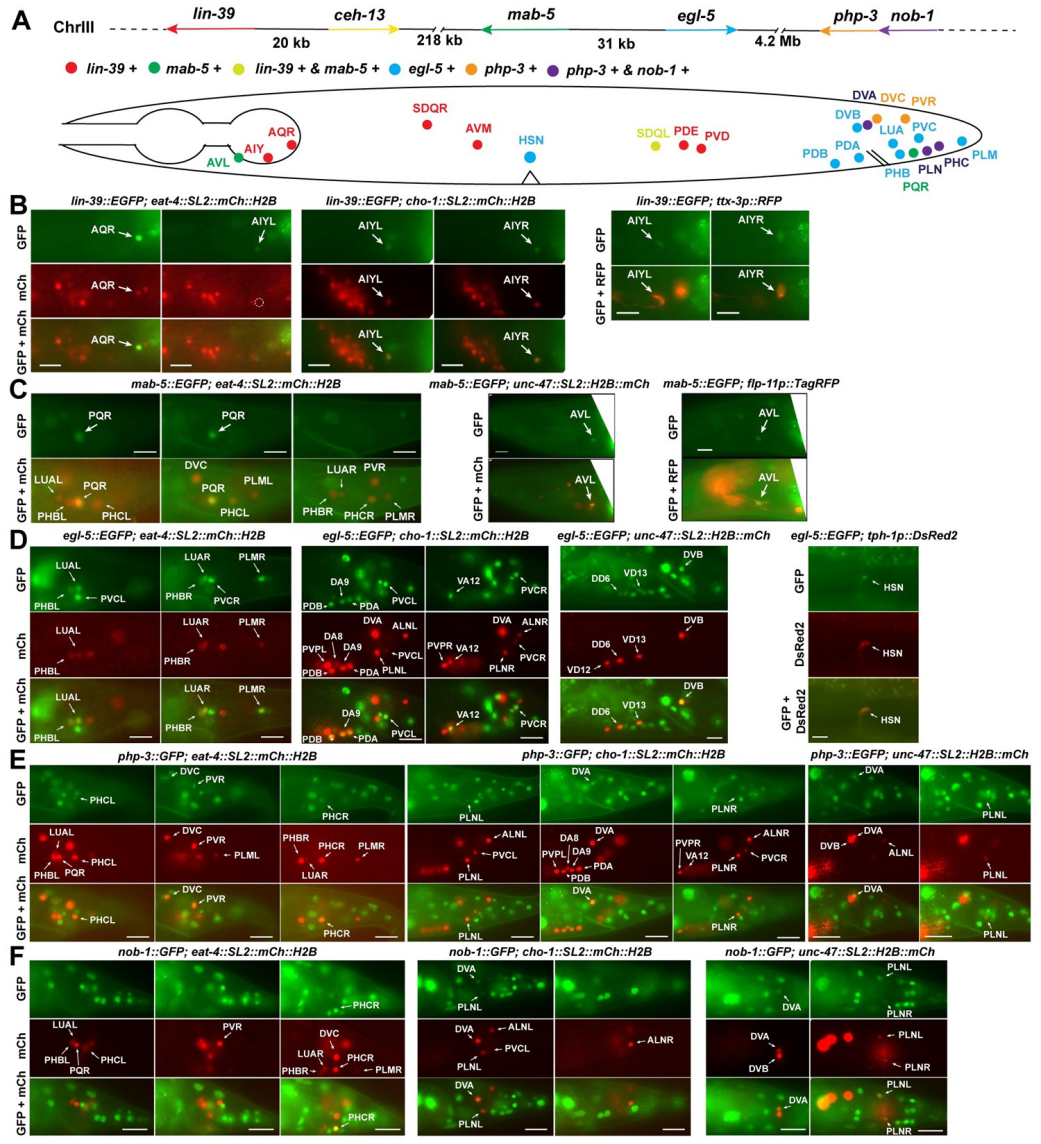
The expression of Hox genes in neurons outside of the ventral nerve cord. (A) The top panel shows the genomic organization of the six Hox genes (color-coded) on chromosome III. The bottom panel shows a summary of Hox expression in neurons outside of the ventral nerve cord MNs. (B) The expression of *lin-39* in AQR and AIY neurons confirmed by the overlapping with the expression of neurotransmitter identity markers, as well as the expression of AIY fate marker *otIs133[ttx-3p*::*RFP]*. Arrows indicate expression and dashed circles indicate no expression. (C) The expression of *mab-5* in PQR and AVL neurons. *unkEx226[flp-11p*::*TagRFP]* served as the fate marker for AVL. (D) The expression of *egl-5* in glutamatergic LUA, PHB, and PLM neurons, cholinergic PDA, PDB, PVC, DA9, and VA12 neurons, GABAergic DVB, DD6, and VD13 neurons, and the serotonergic HSN neurons (marked by *vsIs97[tph-1p*::*DsRed2]*). (E) The expression of endogenous knock-in *php-3*::*GFP(unk25)* in glutamatergic PHC, DVC, and PVR neurons, and cholinergic PLN and DVA neurons. (F) The expression of *nob-1* in PHC, PLN, and DVA neurons. Scale bars = 20 μm.

**Fig 2:**
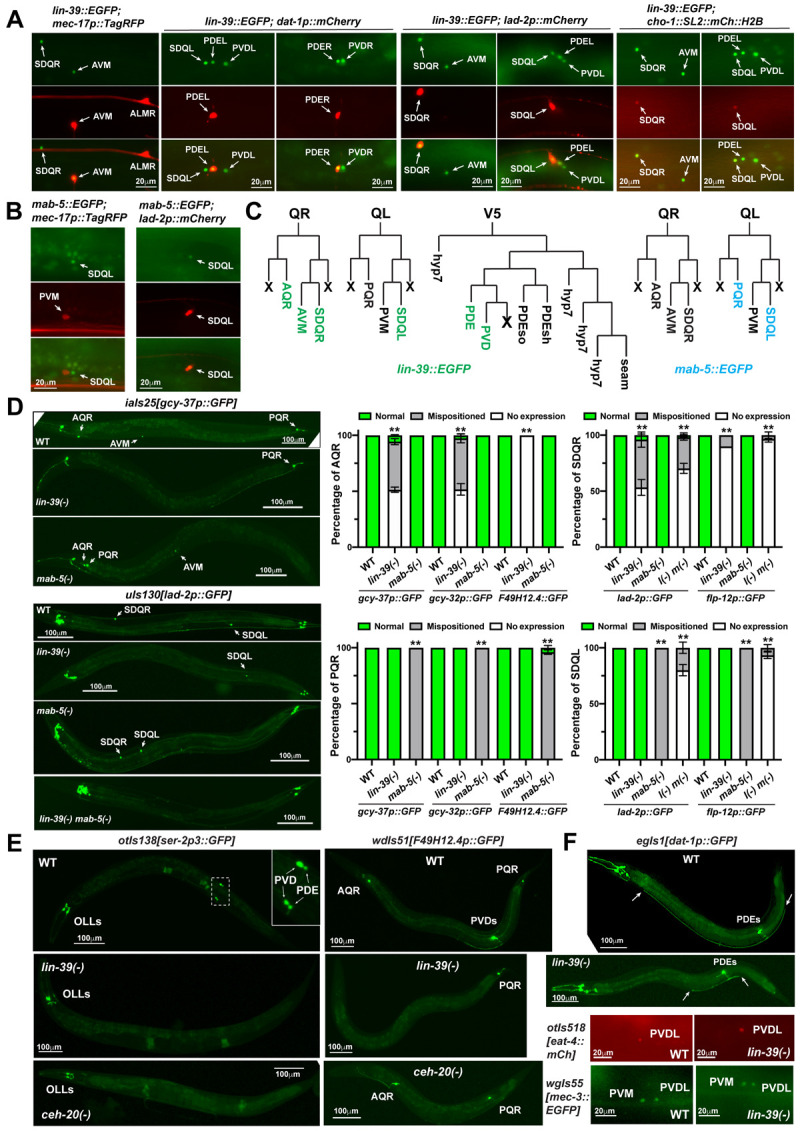
LIN-39 promotes neuronal fate specification in the Q and V5 lineage. (A) The expression of *lin-39* in AVM, SDQL/R, PDEL/R, and PVDL/R, indicated by the overlapping with neurotransmitter identity markers and specific fate markers (*uIs115[mec-17p*::*TagRFP]* for AVM, *otIs181[dat-1p*::*mCh]* for PDE, *uIs117[lad-2p*::*mCh]* for SDQ). (B) The expression of *mab-5* in SDQL. (C) Summary of *lin-39* (green) and *mab-5* (cyan) expression in the descendants of Q and V5 lineages. (D) The loss of *gcy-37* expression in AQR and AVM neurons in *lin-39(n1760)* mutants and the mispositioning of PQR in *mab-5(gk670)* mutants; the loss of *lad-2* expression in SDQR in *lin-39(n1760)* mutants, the displacement of SDQL in *mab-5(gk670)* mutants, and the loss of *lad-2* expression in both SDQs in *lin-39(n1760) mab-5(e1239)* mutants. The right panels show the penetrance for the loss of marker expression and cell body mispositioning. Mean ± SD for the percentage of cells showing corresponding phenotypes from three biological replicates are shown. Double asterisks indicate statistically significant difference (*p* < 0.01) between the mutants and the wild type in a *Chi*-square test. (E) The loss of *ser-2* expression in PVD and PDE neurons and the loss of *F49H12*.*4* expression in PVD in *lin-39(n1760)* and *ceh-20(u843)* mutants. (F) Dopaminergic marker *dat-1* is normally expressed in PDE neurons in *lin-39* mutants, but PDE shows axonal growth defects. The arrows indicate the termini of PDE axons. The expression of glutamatergic identity marker *eat-4* and the PVD terminal selector gene *mec-3* in PVD neurons in *lin-39* mutants.

For *lin-39* and *mab-5*, previous work reported their partially overlapping expression in the ventral cord motor neurons (MNs) [9]. In this study, we used two fosmid reporters *wgIs18[lin-39*::*EGFP]* and *wgIs27[mab-5*::*EGFP]* [12] to map their expression to the single-cell resolution among the 75 motor neurons (MNs), which include 54 cholinergic MNs, 19 GABAergic MNs, and 2 serotonergic MNs with stereotypical positions. These MNs can be further classified into eight neuron classes (DA, DB, VA, VB, AS, VC, DD, and VD). By crossing the GFP reporters with mCherry strains labelling cholinergic or GABAergic neurons in the ventral cord, we identified *lin-39* expression in DA2-5, DB2-7, VA3-8, VB4-9, AS2-8, VC1-6, DD2-6, and VD3-12 (S2 Fig) and *mab-5* expression in DA4-8, DB5-7, VA6-11, VB8-11, AS5-11, VC3-6, DD2-6, and VD2-12 (S3 Fig). Both *lin-39* and *mab-5* expression were generally weaker in more anterior MNs. Interestingly, for cholinergic MNs, *mab-5* and *lin-39* expression only overlapped in a set of mid-body MNs; MNs more anterior to this region expressed only *lin-39* and MNs more posterior expressed only *mab-5*. For GABAergic MNs (DD and VD), however, *lin-39* and *mab-5* expression overlapped entirely. Outside of the ventral nerve cord, *lin-39* was expressed in AQR and AIYL/R in the head and AVM, SDQL/R, PDEL/R, and PVDL/R neurons along the body; *mab-5* was expressed in the AVL in the head, SDQL in the mid-body region, and PQR neuron in the tail (Figs 1A–1C and 2A and 2C).

The other three Hox genes, *egl-5*, *php-3*, and *nob-1*, are homologous to *Drosophila Abd-B* and human *Hox7-12* and were mostly expressed in the tail region. A fosmid reporter *wgIs54[egl-5*::*EGFP]* [12] was expressed in PDA, PDB, DVB, PHBL/R, LUAL/R, PVCL/R, and PLML/R neurons (Fig 1D). Among the MNs, *egl-5* was expressed in the most posterior cell of the DA, VA, DD, and VD types of MNs, namely DA9, VA12, DD6, and VD13. Among them, DA9, VA12, and VD13 did not express either *mab-5* or *lin-39*, while DD6 was the only neuron in the nervous system that expressed three Hox genes, *mab-5*, *lin-39*, and *egl-5*. Outside of the tail region, *egl-5* was also expressed in the terminally differentiated HSN neurons around the vulva (Fig 1D).

*php-3* and *nob-1* genes are next to each other and only separated by 232 bp, suggesting that both genes are regulated by the same sequence upstream of *nob-1* (S4 Fig) and may share similar expression patterns. We found that an endogenous *php-3*::*GFP* knock-in we created via CRISPR/Cas9-mediated gene editing was expressed in DVA, DVC, PVR, PHCL/R, and PLNL/R neurons, whereas a translational reporter *stIs10286[nob-1*::*GFP]* [13] was expressed only in DVA, PHCL/R, and PLNL/R neurons (Fig 1E and 1F), suggesting that *php-3* and *nob-1* may have slightly different expression patterns. Neither *php-3* nor *nob-1* was expressed in the ventral nerve cord. Interestingly, *php-3* and *nob-1* expression and *egl-5* expression were mutually exclusive with no overlapping at all in posterior neurons. These observations indicate that the three posterior Hox genes, although homologous to each other, may have distinct functions in regulating neuronal specification.

### Comparison of Hox expression patterns across different studies

While we were conducting this study, Reilly *et al*. reported the expression of all homeodomain proteins including the six Hox genes and two ParaHox genes (*pal-1* and *vab-7*) [14], and single-cell (sc) transcriptomics data of the *C*. *elegans* nervous system at the L4 stages had also become available [15]. We found that our Hox expression patterns are largely consistent with these published results (S1 Table).

One major discrepancy is that Reilly *et al*. reported *ceh-13* expression in all eight classes of MNs in the ventral nerve cord using the extrachromosomal fosmid reporter *otEx7484[ceh-13*::*EGFP]*, whereas the integrated fosmid reporter *wgIs756* and the endogenous GFP knock-in we used had extremely weak expression in the MNs, which made cell identification difficult. Results from scRNA-seq confirmed the expression of *ceh-13* in seven classes of MNs, supporting Reilly *et al*.’s observations. Thus, we conclude that *ceh-13* may indeed have low level of expression in the MNs, which are below our detection limit.

Expression patterns of *lin-39*, *mab-5*, *egl-5*, *php-3*, and *nob-1* were similar between our results and the other studies (S1 Table), although minor discrepancies existed. For example, we found that *egl-5* was expressed in DD6 and VD13 neurons, but this expression was not identified by the scRNA-seq, which clustered all 13 VD and 6 DD neurons into one VD_DD cluster. *mab-5* expression in DB5-7, VB8-11, and VC3 and VC6 were missed by the scRNA-seq for the same reason. Moreover, although *lin-39* and *mab-5* expression were indeed found in the DA, VA, and AS classes of MNs, the scRNA-seq data did not contain subtype information, because the clustering of the single cell transcriptomic data could only resolve the different MN classes but not the subtypes within each class (except for a few very distinct ones, e.g., DA9, VA12, and VC4_5). Thus, gene expression mapping with fluorescent reporters still provides valuable information.

Using similar mapping approaches, our results agreed with Reilly *et al*.’s results for most parts except for a few cases where the MN subtype expression were disputed (S1 Table). For example, Reilly *et al*. were able to detect *mab-5* expression in many anterior MNs, whereas we found that *mab-5* was mostly expressed in the MNs located in the posterior half of the ventral nerve cord. We reasoned that these anterior expression may be very weak and were not consistently detectable in our analysis. Nevertheless, *mab-5* had clear expression in SDQL (Fig 2B), which was missed by Reilly *et al*. One limitation of our study is that we could not identify neurons with unknown neurotransmitter identity. For example, both Reilly *et al*. (which identified neurons using the newly developed NeuroPAL that labels all neurons with a color map [16]) and scRNA-seq results identified *egl-5* expression in AWA and *php-3* and *nob-1* expression in PVN and PVW. We were not able to identify these expression, as these neurons did not express any of the neurotransmitter identity markers we used.

Since our work, the Reilly *et al*., study, and the scRNA-seq at L4 stage all detected expression in mature and differentiated neurons, we analyzed Hox expression in embryonic neurons using the scRNA-seq data of *C*. *elegans* embryos [17]. In many cases, Hox expression can be detected in the neurons immediately after their generation in the embryos (e.g., *lin-39*, *mab-5*, and *egl-5* expression in the ventral cord MNs, *lin-39* in AIY neurons, *egl-5* in PLM and PHB neurons, *php-3* and *nob-1* in DVA neurons, etc; S2 Table). Since the same expression was also detected at larval and adult stages, we suspect that Hox expression in these neurons are initiated during development and then maintained throughout the lifetime of the animal.

The embryonic scRNA-seq analysis detected *ceh-13* expression in two major embryonic lineages, ABarpp and ABp(l/r)pp, and their decedents. ABarpp lineage gives rise to ADEsh, ALM, BDU, hyp7, and some of the postembryonic H and V lineages (S3 Table). The expression of *ceh-13* in the precursor (ABarppxapp) of ALM and BDU neurons may explain its regulation of ALM differentiation found by previous studies [7], although *ceh-13* expression in differentiated ALM neurons in larva was not detectable. The ABp(l/r)pp lineage gives rise to many neurons, including the ventral cord MNs (e.g., DA, DB, DD) and AIA, RIM, LUA, PVC, PHA, PVP, etc, as well as muscle and epithelium cells. Embryonic scRNA-seq also detected *ceh-13* expression in newly generated DA, DB, and DD neurons (S2 Table), which suggests that *ceh-13* expression in the MNs may be initiated in the embryo and then maintained at low levels throughout larval development.

The other five Hox genes did not show significant expression in early embryonic lineages that give rise to neurons. Since the focus of this study is the activity of Hox genes in regulating terminal neuronal differentiation, we decided to analyze the effects of these five Hox genes on the expression of terminal fate markers of the neurons that showed clear Hox expression.

### LIN-39 regulates the fate specification of AQR, AVM, and SDQ neurons

In the head, *lin-39* is expressed in AIYL/R and AQR neurons, and *mab-5* is expressed in the AVL neuron. We crossed AIY fate markers *mgIs18[ttx-3p*::*GFP]* (*ttx-3* codes for a LIM domain TF) and *otIs123[sra-11*::*GFP]* (*sra-11* codes for a GPCR) into *lin-39(n1760; K210STOP)* null mutation but did not detect any change in AIY marker expression (S4 Table). Similarly. the expression of AVL fate markers were not affected by the *mab-5(gk670)* deletion allele (S4 Table). Thus, LIN-39 and MAB-5 may not be required for the acquisition of AIY and AVL fates, respectively.

AQR, AVM, and SDQR are the descendants of the QR neuroblast; PQR, PVM, and SDQL are the descendants of the QL neuroblast. The four more anterior neurons (AQR, AVM, SDQR, and SDQL) among the six Q descendants expressed *lin-39*::*EGFP* (Fig 1B and 2A–2C). Previous studies reported that LIN-39 is required for the anteriorly directed migration of QR neuroblast [18], but whether *lin-39* affects the expression of terminal neuronal fate markers of QR descendants is unclear. We found that the expression of the A/PQR markers, *iaIs19[gcy-32p*::*GFP]* and *iaIs25[gcy-37p*::*GFP]* (both *gcy-32* and *gcy-37* code for guanylate cyclases), were lost in ~55% of AQR neurons (Fig 2D) and most of the remaining AQR neurons were mispositioned as previously reported [18]. AQR expression of another fate marker *wdIs51[F49H12*.*4p*::*GFP]* (*F49H12*.*4* codes for an uncharacterized protein) was completely lost in *lin-39(-)* mutants (Fig 2E), suggesting that some aspects of the AQR differentiation may be more strongly affected than others by the loss of LIN-39. Because mutations in *lin-39* did not seem to affect cell cycle progression of the Q lineage [19], our data suggest that LIN-39 may be required for the terminal differentiation of the oxygen-sensing neuron fate in AQR neurons.

Interestingly, the acquisition of the same A/PQR fate in the posterior subtype—PQR neurons—did not require regulatory input from Hox genes. PQR neurons did not express *lin-39* but expressed *mab-5*. Mutations in neither *lin-39* nor *mab-5* affected the expression of A/PQR fate markers in PQR (Fig 2A–2D). Nevertheless, PQR neurons in *mab-5(-)* mutants were anteriorly displaced, because *mab-5* is needed for the posterior migration of QL neuroblast [20].

The interneuron SDQL/R and the mechanosensory neuron A/PVM are also derived from the Q lineages. *lin-39* was expressed in both SDQL and SDQR neurons, but the expression of SDQ fate marker *uIs130[lad-2p*::*GFP]* (*lad-2* codes for a L1 cell adhesion molecule) was only lost in SDQR neurons and not affected in the more posterior SDQL in *lin-39(-)* mutants (Fig 2D). The SDQR neurons that still expressed the GFP reporter were posteriorly displaced as expected. Expression of another SDQ marker *ynIs25[flp-12p*::*GFP]* (*flp-12*, as well as *flp-4*, *flp-10*, *flp-11*, and *flp-13* mentioned below, all code for FMRFamide-like peptides) was lost in ~90% of SDQR but remained unaffected in SDQL in *lin-39(-)* mutants (Fig 2D). The more posterior Hox gene *mab-5* was expressed in SDQL but not SDQR; in *mab-5(-)* mutants, we observed the mispositioning of SDQL due to the defect in QL migration, but the SDQ markers were expressed in 100% of the displaced SDQL neurons. Because SDQL neuron expresses both *lin-39* and *mab-5*, we examined the SDQ fate in the *lin-39(-) mab-5(-)* double mutants and found that both *lad-2* and *flp-12* expression were lost in SDQL, suggesting that the two Hox genes act redundantly to induce the SDQ fate in SDQL neurons. Overall, these results indicate that the specification of the SDQ fate requires the Hox genes.

For the postembryonic touch receptor neurons (TRNs), *lin-39* was expressed in AVM and not in PVM. Mutations in *lin-39* led to the posterior displacement of AVM neurons but did not affect the expression of TRN fate markers, such as *zdIs5[mec-4p*::*GFP]* (*mec-4* codes for a DEG/ENaC channel) and *uIs115[mec-17p*::*TagRPF]* (*mec-17* codes for a tubulin acetyltransferase), in the mispositioned AVM neurons (S5 Fig). However, the expression of *gcy-37* (encoding a guanylate cyclase), a subtype-specific marker that was expressed in AVM but no other TRNs, was completely lost in 100% of *lin-39(-)* mutants (Fig 2D). Thus, LIN-39 may contribute to the specification of the AVM identity among the TRN subtypes.

### LIN-39 regulates the terminal differentiation of PVD and PDE neurons

*lin-39* is also expressed in PVD and PDE neurons. We found that the expression of PVD fate markers *otIs138[ser-2p3*::*GFP]* (*ser-2* codes for a serotonin receptor) and *wdIs51[F49H12*.*4p*::*GFP]* were lost in 100% of *lin-39(-)* mutants (Fig 2E). *otIs138[ser-2p3*::*GFP]* also served as a PDE marker and its PDE expression was also lost in *lin-39(-)* mutants. Mutations in *ceh-20*, a Exd/Pbx homolog and Hox co-factor, led to similar loss of PVD and PDE markers (Fig 2E), suggesting that LIN-39 may work with CEH-20 to regulate PVD and PDE development. As a control, mutations in *mab-5* did not affect the expression of PVD and PDE fates (S6A Fig).

Both PDE and PVD are derived from the V5.pa lineages, which also produces two glial cells, PDEso and PDEsh (Fig 2C). The loss of *lin-39* did not affect neurogenesis or gliogenesis in this lineage, because we found that the PDE and PVD neurons in *lin-39(-)* mutants still expressed the neurotransmitter identity markers, *egIs1[dat-1p*::*GFP]* (*dat-1* codes for a dopamine transporter) and *otIs518[eat-4*::*mCherry]* (*eat-4* codes for a vesicular glutamate transporter), respectively, (Fig 2F) and that PDEsh expressed its marker *unkEx83[C34B2*.*9p*::*GFP]* (C34B2.9 codes for an uncharacterized protein) (S6B Fig). In addition, PDE neurons showed shortened axons in *lin-39(-)* mutants (Fig 2F), and PVD dendritic branching was also affected in *lin-39(-)* mutants [21]. These results suggest that LIN-39 mostly regulates terminal differentiation of PVD and PDE neurons and does not control their generation.

Previous studies found that Hox protein EGL-5 promoted the mechanosensory fate specification in PLM neurons through regulating the terminal selector *mec-3*, which in turn controls the expression of terminal fate makers associated with the mechanosensory fate [7]. MEC-3 is also the terminal selector for AVM and PVD neurons [21,22], which expressed *lin-39*. However, the loss of *lin-39* did not affect the expression of *mec-3* in AVM [7] and PVD neurons (Fig 2F), suggesting that LIN-39 may regulate the expression of their fate markers independently of the terminal selector *mec-3*.

### *Cis*-regulatory elements for LIN-39 mediate the regulation of neuronal fate markers

Using LIN-39 as an example, we ask whether Hox proteins control neuronal fate specification by directly regulating the transcription of terminal fate markers. We manually searched for the canonical Hox/Pbx binding motif (TGATNNAT) in the promoter regions of A/PQR, SDQ, and PVD fate marker genes and tested whether these presumably LIN-39-binding sites are required for their expression.

**Fig 3:**
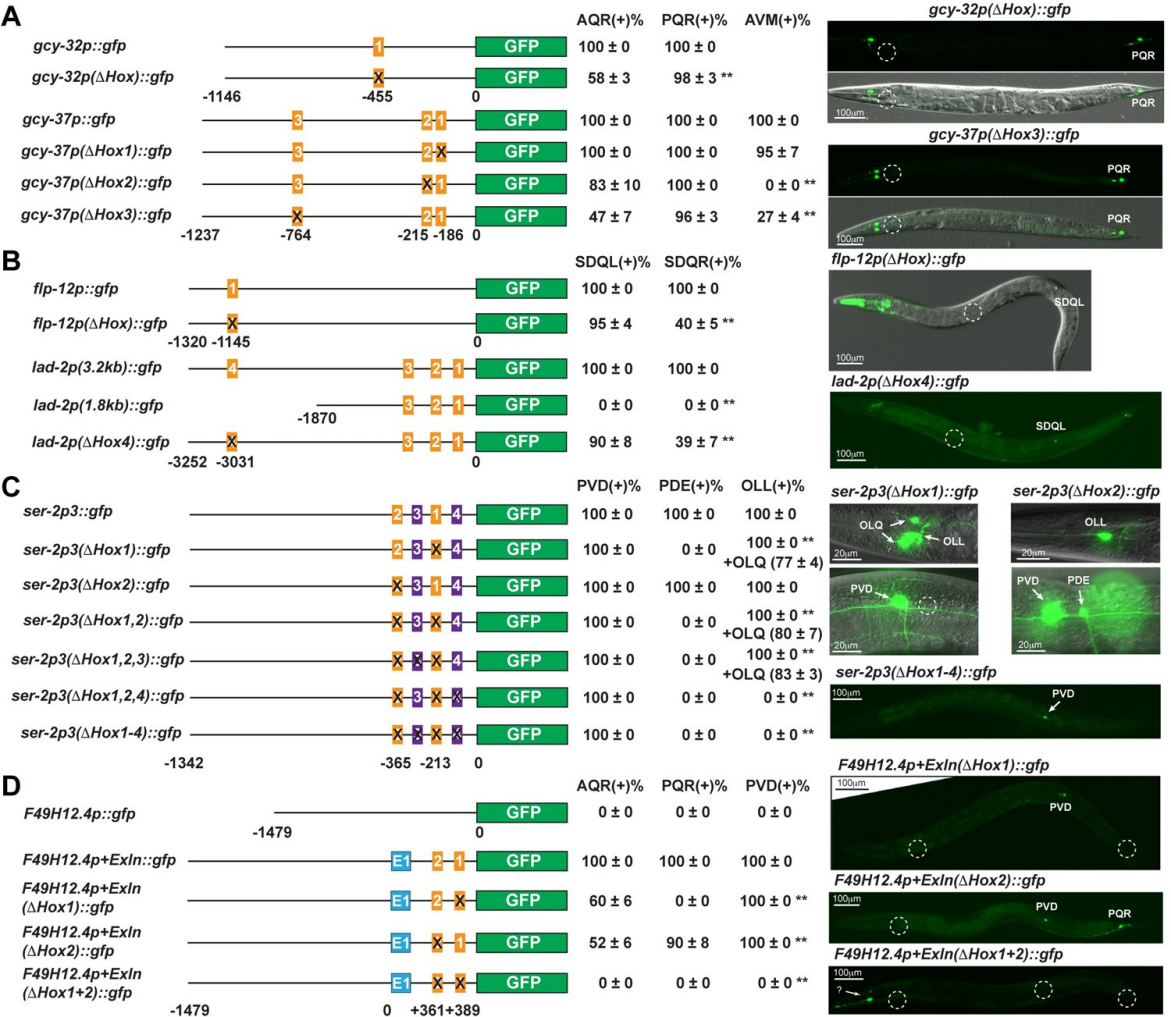
LIN-39 regulates terminal fate markers through Hox binding motifs in their promoters. (A) The effects of deleting Hox sites in *gcy-32* and *gcy-37* promoters on GFP reporter expression in AQR and PQR. The orange boxes represented canonical Hox sites matching the sequence 5’-TGATNNAT-3’ at the indicated position. The circles in the right images indicate the loss of expression. (B) The effects of deleting Hox sites in *flp-12* and *lad-2* promoters on GFP expression in SDQL and SDQR. (C) The effects of deleting Hox sites in *ser-2* promoter. Purple boxes represented alternative Hox sites matching the sequence 5’-TTTG(A/T)AT(T/C)T-3’ at -266 (site #3) and -183 (site #4). (D) The effects of deleting Hox sites in *F49H12*.*4* promoter, which included the first exon (E1) and the first intron (the sequence between E1 and GFP). Removing both sites eliminated expression in AQR, PQR, and PVD but caused ectopic GFP expression in unidentified head neurons (question mark). The quantification shows the mean ± SD for the percentage of cells expressing the GFP from at least two independent lines.

First, deleting the only Hox motif found in *gcy-32* promoter and the Hox site #3 in *gcy-37* promoter specifically affected the expression in AQR neuron, which expressed *lin-39*, but not in PQR neurons, which did not (Fig 3A). The penetrance for the loss of the GFP expression upon the deletion of the Hox sites in the promoters are 40–50%, similar to the penetrance observed in *lin-39(-)* mutants for the wild-type reporters. The expression of *gcy-37* promoter in AVM depended on both Hox site #2 and #3 (Fig 3A).

Second, for SDQ marker *flp-12*, deleting the only Hox motif caused the loss of GFP expression in 60% of SDQR neuron but did not affect expression in SDQL neuron (Fig 3B). Similar results were found when Hox site #4 was deleted in the promoter of another SDQ marker gene *lad-2*. These results were consistent with the finding that *lin-39(-)* mutants only showed fate specification defects in SDQR but not in the more posterior SDQL. The normal SDQL expression of the reporter without the Hox site suggests that the same site is not used by MAB-5 for activating SDQ markers, given that LIN-39 and MAB-5 act redundantly in SDQL.

Third, for PVD and PDE marker *ser-2*, we found that the Hox site #1 was required for PDE expression, but the *ser-2* expression in PVD neurons was not affected when both canonical Hox site #1 and #2 were deleted (Fig 3C). Deleting site #1 led to dereprerssion in OLQ neurons. We then searched for sequences that matched an alternative LIN-39 binding motif TTTG(A/T)AT(T/C)T identified by Roiz *et al*. [23] and found two such sites (#3 and #4). Nevertheless, deleting all four Hox sites (#1~4) still did not affect PVD expression, suggesting that LIN-39 may regulate the PVD expression of *ser-2* indirectly.

Finally, *F49H12*.*4* served as the fate marker for both A/PQR and PVD neurons. We found two canonical Hox sites in the first intron of *F49H12*.*4*. Removing site #2 led to an AQR-specific loss of marker expression, similar to the phenotype in *lin-39(-)* mutants (Fig 3D). Removing site #1, however, affected *F49H12*.*4* expression in both AQR and PQR, with a more severe loss in PQR. Thus, site #2 may be used by LIN-39 for AQR-specific activation of *F49H12*.*4*, whereas site #1 is a more general *cis*-regulatory element for A/PQR fate. Although deleting site #1 and site #2 alone did not affect PVD expression, removing both led to complete loss of marker expression in PVD neurons.

The functional Hox sites identified in the above promoters resembled the LIN-39 motif M09582_2.00 in the CIS-BP database (S7 Fig), which was derived from ChIP-seq results [12]. In fact, we observed clear LIN-39 binding to these Hox sites in the modEncode ChIP-seq data [12], and the binding signal was especially strong for *ser-2*, *flp-12*, and *F49H12*.*4* promoters (S8A Fig). The ChIP-seq signal in *ser-2* promoter likely reflects LIN-39 binding to *ser-2* in PDE but not PVD neurons. These results suggest that canonical Hox sites mediate the activation of terminal fate markers presumably by recruiting Hox proteins (e.g., LIN-39) expressed in the neurons.

### EGL-5 regulates fate specification of several tail neurons

**Fig 4:**
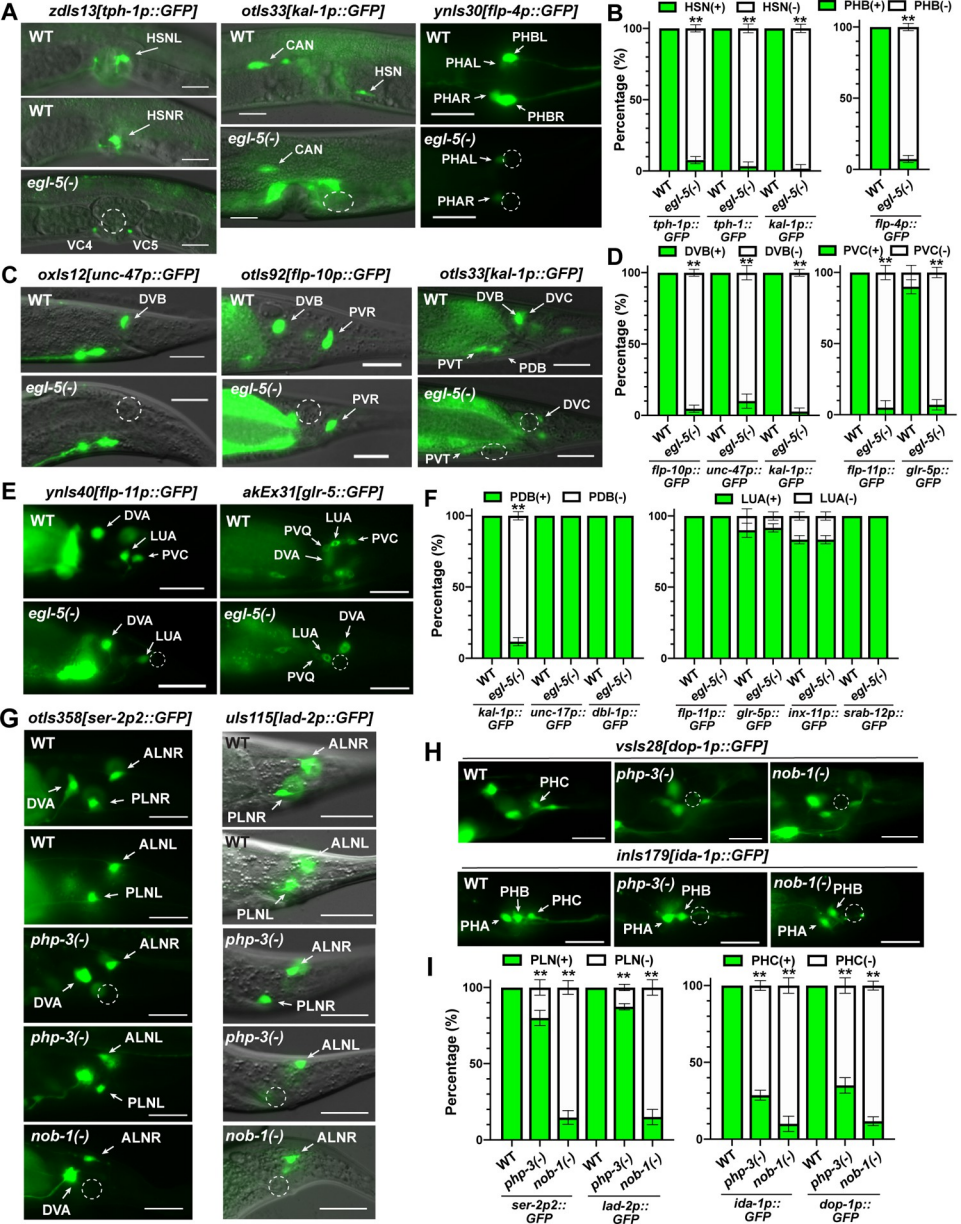
EGL-5, PHP-3, and NOB-1 regulates fate specification of tail neurons. (A) The expression of fate markers *tph-1* and *kal-1* were lost in HSN in *egl-5(u202)* mutants; *tph-1* was ectopically expressed in VC4 and VC5 neurons. Expression of PHB fate marker *flp-4* was lost in *egl-5* mutants. (B) The penetrance of the HSN and PHB marker expression; *tph-1p*::*GFP* and *tph-1*::*GFP* show the results of *zdIs13* and *mgIs42* expression, respectively. (C) The loss of fate markers *unc-47*, *flp-10*, and *kal-1* in DVB neurons in *egl-5* mutants; *kal-1* expression was also lost in PDB neurons. (D) The penetrance of DVB and PVC marker expression. (E) The loss of PVC fate marker *flp-11* and *glr-5* expression in *egl-5* mutants. (F) The penetrance of PDB and LUA marker expression. (G) The loss of *ser-2* and *lad-2* expression in PLN neurons in *php-3(ok919*) and *nob-1(ct230)* mutants. (H) The loss of *dop-1* and *ida-1* expression in PHC neurons in *php-3* and *nob-1* mutants. (I) The penetrance of PLN and PHC fate marker expression. Scale bars = 20 μm. Quantification shows mean ± SD for the percentage of cells showing expression from three biological replicates. Double asterisks indicate statistically significant difference (*p* < 0.01) between the mutants and the wild type in a *Chi*-square test.

Among the neurons that expressed *egl-5*, previous studies had shown that *egl-5* is required for the differentiation of HSN, PDA, PLM, and PVC fate [6–8,24]. PHB neuron is the sister cell of HSN, and *egl-5* is expressed in the HSN/PHB precursor, as well as the two terminally differentiated neurons. Expression of the HSN markers *zdIs13[tph-1p*::*GFP]*, *mgIs42 [tph-1*::*GFP]*, and *otIs33[kal-1p*::*GFP]* (*tph-1* codes for a tryptophan hydroxylase and *kal-1* codes for an extracellular matrix glycoprotein) were lost in ~90% of *egl-5(-)* mutants (Fig 4A and 4B), which is consistent with the role of *egl-5* in regulating the migration and maturation of HSN neurons [8]. Interestingly, in the absence of proper HSN differentiation, *egl-5(-); zdIs13* animals showed misexpression of *tph-1* in VC4 and VC5 neurons around the vulva (Fig 4A).

Although the loss of *egl-5* alone did not affect the expression of PHB marker *gmIs13[srb-6*::*GFP]* (*srb-6* codes for a GPCR), mutations in *egl-5* significantly enhanced the loss of PHB marker expression in the *ham-1(-)* background, suggesting that EGL-5 contributed to the specification of PHB fate [25]. In this study, we found that another PHB marker *ynIs30[flp-4p*::*GFP]* was lost in ~88% of PHB neurons in *egl-5(-)* mutants (Fig 4A and 4B). It is also noteworthy that Singhvi *et al*., (2008) reported that *egl-5* was not expressed in PHB neurons using *muIs13[egl-5*::*lacZ]*, whereas we found that the *wgIs54[egl-5*::*EGFP]* fosmid reporter was indeed expressed in PHB neurons (Fig 1). This PHB expression was also identified by scRNA-seq data [15] and the Reilly *et al*. study [14]. So, our results support the idea that EGL-5 promotes PHB differentiation.

*egl-5* is also essential for the differentiation of DVB neurons, which are derived from transdifferentiation of the K rectal epithelial cell [26]. Three DVB markers *otIs92[flp-10p*::*GFP]*, *oxIs12[unc-47p*::*GFP]* (*unc-47* codes for a vesicular GABA transporter), and *otIs33[kal-1p*::*GFP]* were all lost in >95% of *egl-5(-)* mutants (Fig 4C and 4D). *ynIs40[flp-11p*::*GFP]* was previously identified as a DVB marker [27], but we found that *flp-11* was actually expressed in DVA neurons (by overlapping with neurotransmitter identity markers). *flp-11* expression in DVA was not affected by mutations in *egl-5*, which is not expressed in DVA. Nevertheless, *flp-11* expression in PVC was abolished in *egl-5(-)* mutants (Fig 4E).

*egl-5* is also expressed in the PDB and LUA neurons. We found that the expression of PDB marker *otIs33[kal-1p*::*GFP]* was lost in most *egl-5(-)* mutants, but the expression of a general cholinergic marker *vsIs48[unc-17p*::*GFP]* (*unc-17* codes for a vesicular acetylcholine transporter) and a motor neuron marker *ctIs43[dbl-1p*::*GFP]* (*dbl-1* codes for a BMP-like protein) were not affected (Fig 4C and 4F). So, EGL-5 may contribute to some aspects of PDB fate specification. None of the LUA markers we examined, including *ynIs40[flp-11p*::*GFP]*, *akEx31[glr-5*::*GFP]*, *zwEx111[inx-11p*::*GFP]*, and *sIs12174 [srab-12p*::*GFP]* (*glr-5* codes for an AMPA glutamate receptor, *inx-11* codes for a gap junction protein, and *srab-12* codes for a GPCR) showed any difference in expression between the wild-type and *egl-5(-)* animals (Fig 4E and 4F). The expression of another LUA marker *lhIs97[plx-2p*::*mCherry]* was not affected by mutations in *egl-5* either, according to Kurland *et al* [28]. Thus, *egl-5* regulates the specification of six neuronal fates (PDA, PDB, DVB, PHB, PVC, and PLM) and is dispensable for the specification of one neuronal fate (LUA) in the tail region.

Moreover, to examine whether EGL-5 directly regulates the terminal fate markers of the tail neurons studied above, we analyzed the EGL-5 ChIP-seq results in the modEncode dataset. We found clear EGL-5 binding signals in the promoters of *kal-1*, *glr-5*, *flp-4*, *flp-10*, and *unc-47* (S8B Fig), suggesting that EGL-5 may directly regulate their transcription.

### PHP-3 and NOB-1 regulate PHC and PLN differentiation

In addition to *egl-5*, two other posterior (*Abd-B*-related) Hox genes, *php-3* and *nob-1*, are both expressed in the tail neurons DVA, PHC, and PLN, which did not express *egl-5*. Mutations in either *php-3* or *nob-1* affected the expression of PHC fate markers, *inIs179[ida-1p*::*GFP]* and *vsIs28[dop-1*::*GFP]* (*ida-1* codes for a type N protein tyrosine phosphatase and *dop-1* codes for a dopamine receptor), and the expression of PLN markers, *uIs130[lad-2p*::*GFP]* and *otIs358[ser-2p2*::*GFP]* (Fig 4G and 4H). These results suggest that *php-3* and *nob-1* are required for the fate specification of PHC and PLN. As controls, the specification of PHA, PHB, and ALN fates were not affected in the Hox mutants.

*nob-1* appeared to play a bigger role than *php-3* in regulating PHC and PLN differentiation as *nob-1(-)* mutants showed higher percentage of cells without the marker expression (Fig 4I). This finding is consistent with previous report that *nob-1(-)* mutants had higher penetrance of tail formation defects than *php-3(-)* mutants and *nob-1* was expressed at higher level than *php-3* [29]. In our hands, we found that ~15% of *nob-1(-)* mutants showed severe deformation in the posterior region and they were excluded from our analysis of terminal neuronal differentiation; in contrast, no *php-3(-)* mutants showed tail deformation.

Although *php-3* and *nob-1* are expressed in DVA neurons, mutations in neither of them affected the expression of the DVA markers, including *otIs358[ser-2p2*::*GFP]*, *ctIs43[dbl-1p*::*GFP]*, and *ynls40[flp-11p*::*GFP]* (S9A Fig). We could not test the redundancy between *php-3* and *nob-1*, because the *php-3(-) nob-1(-)* double mutants resulted in 100% larval lethality and showed no posterior morphogenesis [29]. *php-3* was also expressed in DVC and PVR neurons that did not express *nob-1*, but the expression of DVC fate marker *sIs12174 [srab-12p*::*GFP]* and PVR fate marker *otIs92[flp-10*::*GFP]* did not show any defects in *php-3(-)* mutants (S9B Fig). As expected, mutations in *nob-1* did not affect DVC and PVR fate either.

### LIN-39, MAB-5, and EGL-5 regulate motor neuron subtype diversification

**Fig 5:**
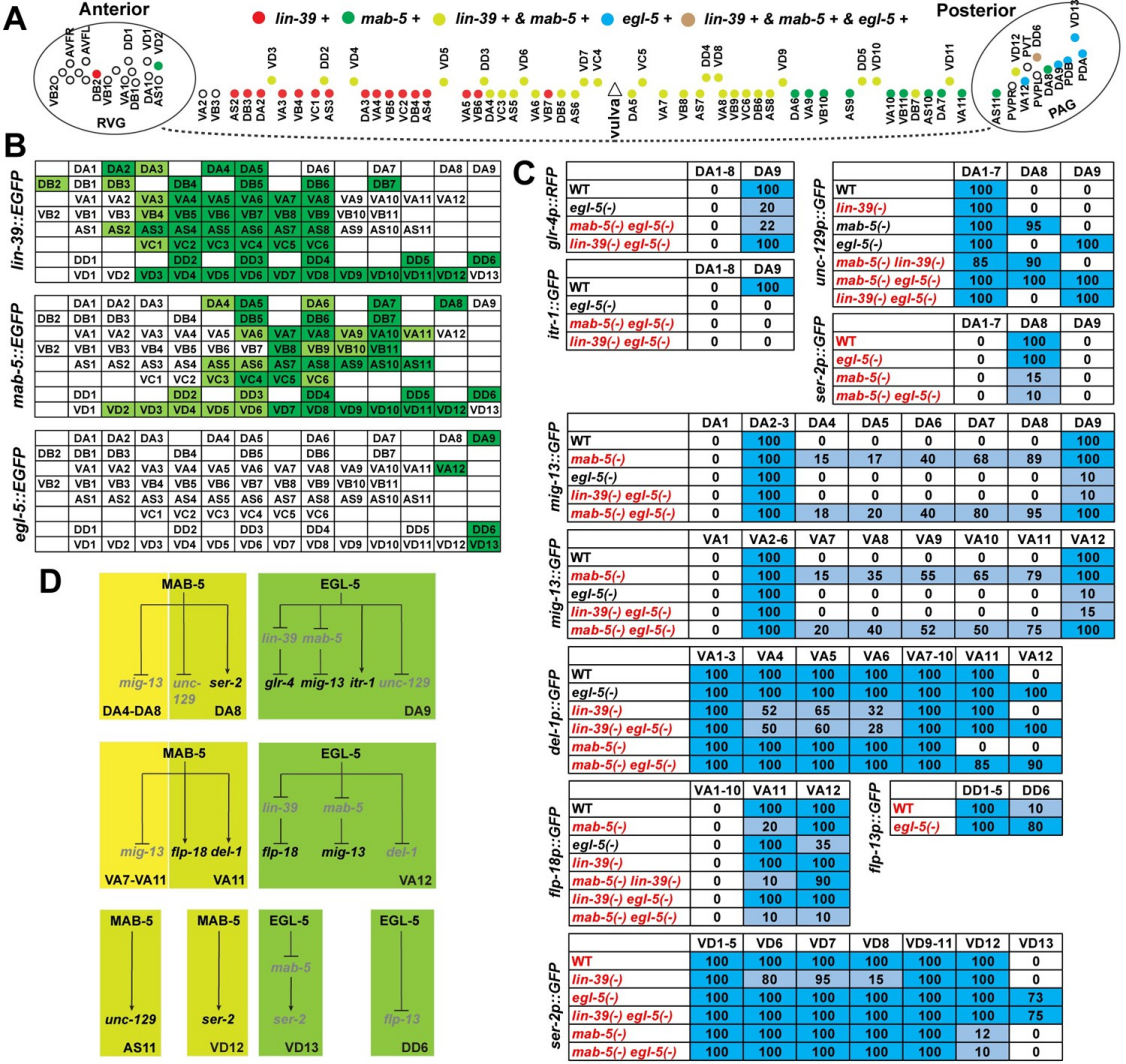
The expression of Hox genes in MNs and their regulation of MN subtype diversification. (A) A summary of *lin-39*, *mab-5*, and *egl-5* expression in cholinergic and GABAergic MNs in the ventral nerve cord. (B) The expression of the three Hox genes among the subtypes of the eight types of MNs. Dark and light green indicates strong and weak expression of the reporters, respectively. (C) Summary of the subtype marker expression in wild-type animals and Hox mutants. The number in the tables indicate the percentage of cells expressing the corresponding GFP reporter; the mean of three biological replicates is shown (raw results in S6 Table). Genotype names in black indicate our confirmation of previous results [9] and red names indicate new results from this study. Dark blue, light blue, and white highlighting indicate highly penetrant, reduced or ectopic, and no expression, respectively. (D) A model for the activity of MAB-5 and EGL-5 in regulating the molecular features of the MN subtypes. Genes in black are expressed and genes in light grey are not expressed.

A majority of the neurons that express the middle-group Hox genes *lin-39* and *mab-5* are ventral nerve cord motor neurons (MNs). Their expression domains partially overlapped, showing *lin-39* expression in more anterior MNs and *mab-5* in more posterior MNs (Figs 5A, 5B and S2–S3). Nevertheless, their expression did not cover three of the most posterior MNs, namely DA9, VA12, and VD13, which instead expressed a posterior-group Hox gene, *egl-5* (Fig 1D). This differential Hox expression along the anterior-posterior axis immediately suggests a Hox-mediated mechanism of MN subtype diversification.

**Fig 6:**
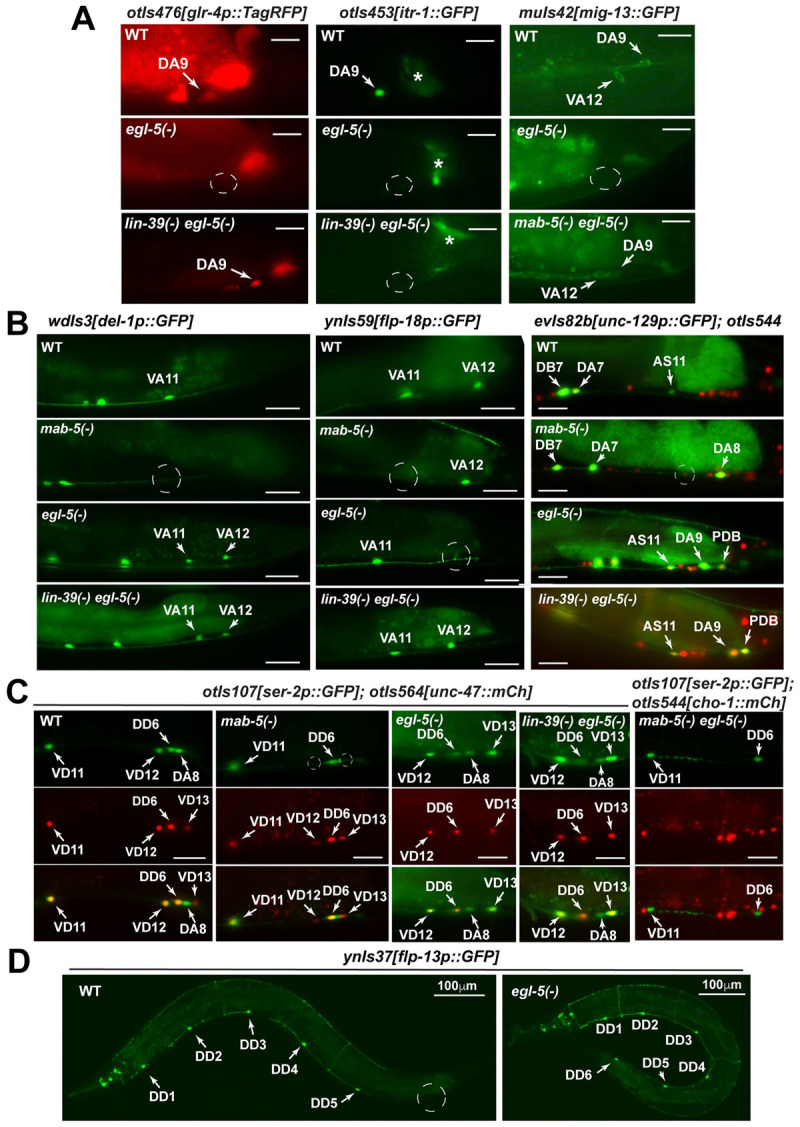
The regulation of MN subtype markers by Hox genes. (A) The loss of *glr-4*, *itr-1*, and *mig-13* expression in DA9 and the loss of *mig-13* expression in VA12 in *egl-5(u202)* mutants. *glr-4* and *mig-13* expression were restored in *lin-39(n1760) egl-5(n945)* and *mab-5(e1239) egl-5(n945)* double mutants, respectively. (B) The loss of *del-1* and *flp-18* expression in VA11 in *mab-5(gk670)* mutants. Ectopic *del-1* expression and loss of *flp-18* expression in VA12 in *egl-5* mutants. Derepression of *unc-129* in DA8 and DA9 in *mab-5* and *egl-5* mutants, respectively. *unc-129* expression was also lost in AS11 in *mab-5* mutants and derepressed in PDB in *egl-5* mutants. (C) *ser-2* expression was lost in VD12 and DA8 in *mab-5* mutants and derepressed in VD13 in *egl-5* mutants. Scale bars = 20 μm in (A-C). (D) Ectopic expression of *flp-13* in DD6 in *egl-5* mutants.

Previous studies identified several subtype markers among the cholinergic MNs and found that Hox genes contribute to their expression [9], but the mechanism is not fully understood. In this study, by comparing the expression of subtype markers in Hox single and double mutants, we ask whether Hox genes regulate the expression of subtype markers directly or indirectly. For example, among the nine DA-type MNs, the most posterior DA9 specifically expressed *otIs476[glr-4p*::*TagRFP]* (*glr-4* codes for an AMPA glutamate receptor) and *otIs453[itr-1p*::*GFP]* (*itr-1* codes for an inositol triphosphate receptor); these DA9-specific expressions were lost in *egl-5(-)* mutants (Figs 5C and 6A). However, *glr-4* expression was restored in *lin-39(-) egl-5(-)* double mutants but not in *mab-5(-) egl-5(-)* double mutants. *itr-1* expression was not restored in either double mutant, suggesting that EGL-5 activated *glr-4* through repressing *lin-39* and activated *itr-1* independently of *lin-39* and *mab-5*. Similarly, EGL-5 activated *mig-13* (which codes for a Frizzled receptor) through repressing *mab-5* and repressed *unc-129* (which codes for a bone morphogenetic protein) independently of *mab-5* and *lin-39* in DA9 neurons (Fig 6A and 6B). Given that the promoters of these MN subtype markers all contain functional Hox binding sites [9], Hox proteins may directly regulate their transcription. The above results suggested that EGL-5 induced DA9 specification by both repressing more anterior Hox genes and directly activating downstream genes.

In addition to EGL-5, MAB-5 also specifies MN subtypes in its expression domain. For example, the expression of *mig-13* was found in the DA2, DA3, and DA9 but not in the DA4-8 neurons that expressed *mab-5*. Expression of *mig-13* was derepressed in *mab-5(-)* mutants in the middle-body DA neurons (Figs 5C and S10A), suggesting that MAB-5 actively repressed *mig-13* to cause molecular diversification of DA neurons.

MAB-5 induced DA8 differentiation into a specific DA subtype based on the expression of subtype markers. A boundary between DA7 and DA8 was observed for *unc-129* expression, which was found in DA1-7 but not in DA8-9. Loss of *mab-5* leads to the derepression of *unc-129* in DA8, similar to its derepression in DA9 in *egl-5(-)* mutants (Figs 5C and 5D and 6B). Thus, MAB-5 and EGL-5 carried out similar functions in repressing *unc-129* to specify DA8-9. Moreover, DA8 expressed *ser-2* (which codes for a serotonin receptor) exclusively among the nine DA neurons, and this expression depended on *mab-5* (Figs 5C and 5D and 6C). The DA7-to-DA8 boundary cannot be explained by Hox expression alone, since both DA7 and DA8 express only one Hox gene *mab-5*. Other factors like cell-extrinsic cues may also contribute to DA8 specification.

Besides DA-type MNs, we made similar observations in VA-type MNs. For example, EGL-5 repressed *del-1* (which codes for an epithelial sodium channel) and activated *mig-13* in VA12 through repressing *mab-5* (Fig 6A and 6B). In VA7-VA11, MAB-5 repressed the expression of *mig-13*, carving out a subtype identity for the middle-body VA neurons. Similar to DA neurons, a boundary between VA10 and VA11 also existed; *mab-5* activated *del-1* in VA11, but *del-1* expression in more anterior VAs (VA6-10) did not require *mab-5*. Expression of *flp-18* was detected in VA11-12 but not VA1-VA10 neurons, and its expression depended on *mab-5* and *egl-*5 in VA11 and VA12, respectively (Figs 5C and 6B). These results suggest that the most posterior DA and VA neurons that expressed *mab-5* (i.e. DA8 and VA11) acquired MAB-5-dependent molecular features and differentiated into distinct subtypes. Supporting this hypothesis, the most posterior AS neuron that expressed *mab-5* (i.e. AS11) also defined a AS subtype by specifically expressing *unc-129* among the eleven AS neurons (Fig 6B). As expected, its expression was lost in *mab-5(-)* mutants.

Not only for cholinergic MNs, GABAergic MNs also showed Hox gene-dependent diversification along the A-P axis. EGL-5 inhibited the expression of *ser-2* by repressing *mab-5* in VD13, whereas VD1-12 expressed *ser-2* normally (Figs 5C and 6C). In VD12, the most posterior VD neuron expressing *mab-5*, the expression of *ser-2* required *mab-5*. The above results suggest that EGL-5 and MAB-5 regulate subtype diversification within the same type of MNs by controlling gene expression in the most posterior and the penultimate neurons, respectively (Fig 5D).

In DD-type GABAergic MNs, we identified a DD1-5-specific subtype marker *flp-13*, which has no or very weak expression in DD6 (Fig 6D). All six DD neurons expressed *lin-39* and *mab-5*, but *egl-5* was expressed only in DD6 and not DD1-5. The loss of *egl-5* led to the derepression of *ynIs37[flp-13p*::*GFP]* in DD6 neurons (Figs 5C and 6D), suggesting that EGL-5 also functions to diversify the DD MNs along the A-P axis.

LIN-39 appeared to play a relatively smaller role in regulating MN subtype diversification, since *lin-39* is generally not required for the expression of subtype markers for the anterior MNs. For example, the DA1-7 expression of *unc-129* was not affected in *lin-39(-)* mutants, although DB2-4 expression of *unc-129* had variable loss (S10B Fig); the VA1-11 expression of *del-1* was mostly normal in *lin-39(-)* mutants except for ~50% of loss in VA4-6 neurons (S10C Fig). Nevertheless, Kratsios *et al*. reported that fewer DA/DB and VA/VB neurons expressed *unc-129* and *del-1*, respectively, in *lin-39 mab-5* double mutants, compared to the wild-type animals [9], which suggested genetic redundancy between *lin-39* and *mab-5* presumably in the neurons that expressed both (DA4-5, DB5-7, VA6-8, and VB8-9). In addition, we found that *mig-13* expression in DA2-3 and VA2-6 were not affected by mutations in *lin-39*; and VD1-12 expression of *ser-2* were mostly maintained in *lin-39(-)* mutants except for variable loss of expression in VD6-8 (S10D Fig).

The expression of the subtype markers of cholinergic MNs were, however, dependent on the terminal selector UNC-3, a COE transcription factor [9], suggesting that terminal selectors of general MN fates also serve as the activators of subtype markers. In the GABAergic MNs, the expression of the subtype marker *flp-13* for DD neurons depends on the terminal selector UNC-30, a homeodomain transcription factor [30]. Interestingly, we found that the subtype marker *ser-2* for VD neurons appeared to be mostly independent of UNC-30 (S10D Fig), suggesting the possible existence of other regulators for GABAergic MN fate.

### Cross-regulation among Hox genes in MNs and EGL-5-induced repression of TALE cofactors in VD13

**Fig 7:**
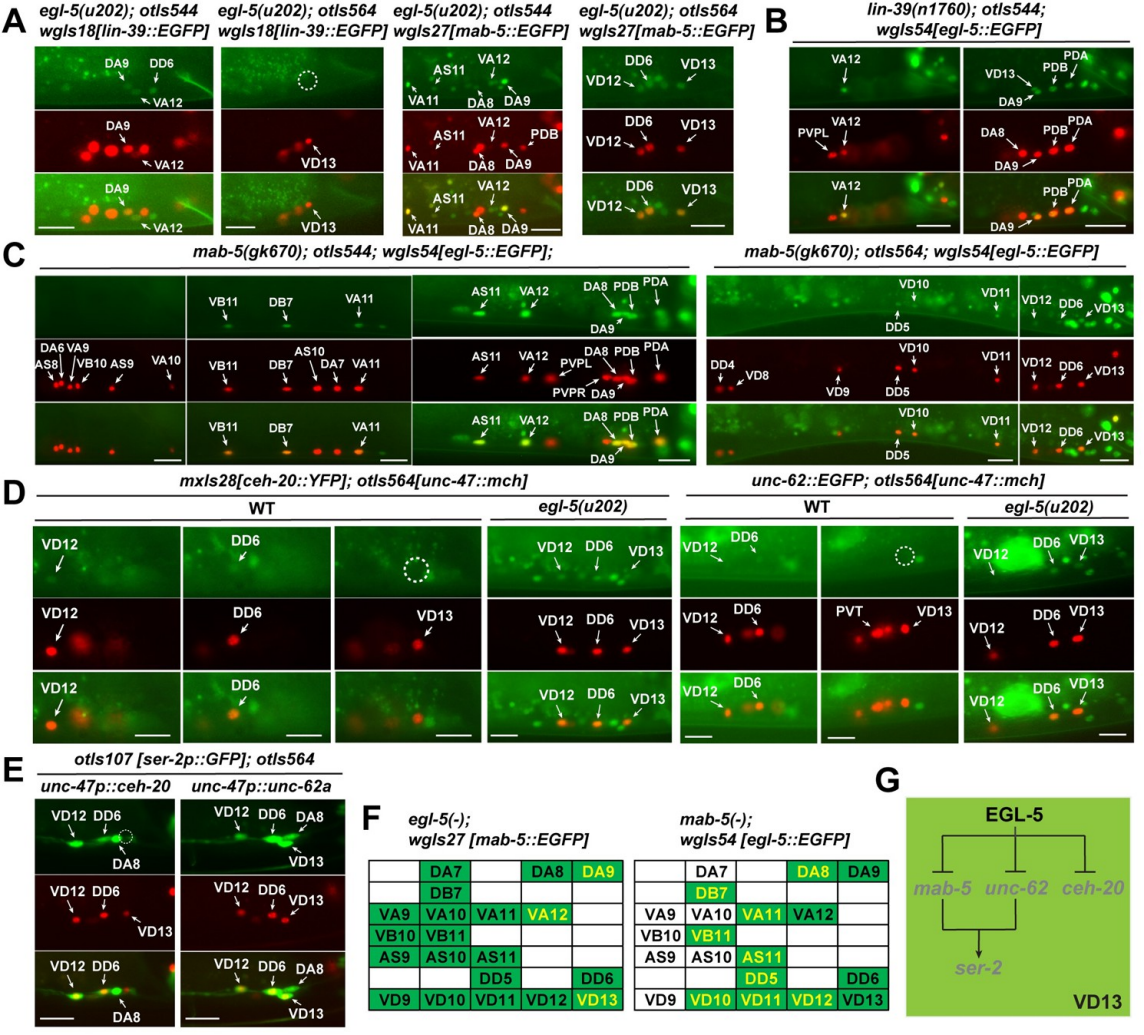
Cross regulation of Hox genes and the repression of TALE cofactors by EGL-5. (A) Ectopic expression of *lin-39* in DA9 and VA12 but not VD13 and ectopic expression of *mab-5* in DA9, VA12, and VD13 in *egl-5* mutants. (B) *egl-5* expression pattern is not changed by mutations in *lin-39*. (C) Ectopic expression of *egl-5* in cholinergic VB11, DB7, VA11, AS11, and DA8 and GABAergic DD5 and VD10-12 in *mab-5* mutants. (D) Derepression of *ceh-20* and *unc-62* in VD13 neurons in *egl-5(u202)* mutants. (E) Ectopic expression of *ser-2* in VD13 upon the misexpression of the *a* isoform of *unc-62*. (F) A summary of cross regulation between *egl-5* and *mab-5*. Cells that show ectopic Hox expression in the mutants of the other Hox gene are in yellow. (G) The model for EGL-5 repressing the VD subtype marker *ser-2* in VD13 through repressing anterior Hox gene *mab-5* and the Meis cofactor *unc-62*. Scale bars = 20 μm.

Because the Hox genes showed distinct expression domains along the ventral nerve cord, we next studied how Hox genes regulated each other in the MNs. First, *lin-39* was derepressed in DA9 and VA12 and *mab-5* was derepressed in DA9, VA12, and VD13 in *egl-5(-)* mutants (Fig 7A). These results were consistent with the findings that EGL-5 indirectly regulate subtype markers through repressing *lin-39* and *mab-5* in these neurons.

Second, the expression of *egl-5* was not affected by the loss of *lin-39* (Fig 7B), but *egl-5* was derepressed in more anterior MNs in *mab-5(-)* mutants, including DA8, DB7, VA11, VB11, AS11, DD5, and VD10-12 neurons (Fig 7C and 7F). The reciprocal inhibition between *mab-5* and *egl-5* suggests that the establishment of specific Hox zones in MNs along the A-P axis not only relies on posterior dominance but also depends on the anterior Hox genes repressing the immediately posterior one.

In addition to the Hox genes, we also mapped the expression of two important TALE-class Hox cofactors, CEH-20/Pbx and UNC-62/Meis, in the MNs and found that both *ceh-20* and *unc-62* were expressed in almost all MNs, except VD13 and a few anterior MNs, which suggests that the cofactors may work with both LIN-39 and MAB-5. Interestingly, both *ceh-20* and *unc-62* were derepressed in VD13 in *egl-5(-)* mutants, suggesting that *egl-5* actively repressed the expression of Hox cofactors in VD13 (Fig 7D).

Since EGL-5 controlled the VD13-specific repression of *ser-2* among the VD neurons, we next tested whether EGL-5 did so through the repression of the Hox cofactors. Ectopic expression of *unc-62* and not *ceh-20* in all VD neurons using the *unc-47* promoter activated *ser-2* in VD13, making it molecularly more similar to VD1-12 (Fig 7E and 7G). These results suggest that not only EGL-5 works independently of the cofactors, but the presence of the UNC-62 cofactor disrupted the function of EGL-5 in regulating target genes. The findings in VD13 are similar to previous results in the PLM neurons, where EGL-5 also repressed the expression of TALE cofactor and forced expression of *unc-62* blocked the activity of EGL-5 in inducing PLM features [6]. Similar relationships between the posterior Hox genes and TALE cofactors were also observed for fly *Abd-B* [31] and mouse *Hoxa10* [32].

## Discussion

### A summary of Hox activities in specifying terminal neuronal fates in the nervous system

In this study, we first generated a Hox expression map in *C*. *elegans* nervous system and then analyzed whether and how Hox genes regulated the terminal differentiation of the neurons that expressed the Hox genes. Except for *ceh-13/Hox1*, which is mainly expressed in the embryos, the other five Hox genes (*lin-39/Hox4-5*, *mab-5/Hox6*, *egl-5/Hox7-8*, *php-3/Hox10*, and *nob-1/Hox12*) were expressed in 97 (32%) of the 302 neurons in adult hermaphrodites. These 97 neurons fall into 29 (25%) neuron types or classes out of the 118 anatomic classes previously defined [33]. Majority (71) of the Hox-expressing neurons are motor neurons (covering 13 of the total 32 motor neuron types); 15 are sensory neurons (covering 9 of the 38 sensory neuron types); 11 are interneurons (covering 7 of the 48 interneuron types).

Among the 29 types of neurons that express Hox genes, this study and previous work together found direct evidence for Hox regulation of terminal fate specification in 22 neuron types, covering 19% of the nervous system in neuron types (Table 1). These findings suggest that a small number (five) of Hox genes contribute to the differentiation of many neuronal cell fates. Although we expect these Hox genes to exert their regulatory function during terminal differentiation, we could not rule out the possibility that they also control neurogenesis in some cases (asterisks in Table 1). We could do so, however, in cases that only some of the fate markers were affected in the Hox mutants, while other unaffected markers could label the presence of the cell.

**Table 1:** A summary of the Hox regulation of neuronal differentiation in the *C*. *elegans* nervous system.

		Hox regulation	Reference
**DA**	Motor neuron	LIN-39 and MAB-5 regulate *unc-129* in DA2-8; MAB-5 represses *mig-13* in DA4-8 and activates *ser-2* in DA8; EGL-5 induces *glr-1* and *itr-1* in DA9	[9] and this study
**DB**	Motor neuron	LIN-39 and MAB-5 regulate *unc-129* in DB2-7	[9]
**AS**	Motor neuron	MAB-5 induces *unc-129* in AS11	This study
**VA**	Motor neuron	LIN-39 and MAB-5 regulate *del-1* in VA3-11; MAB-5 represses *mig-13* in VA7-11 and induces *flp-18* in VA11; EGL-5 represses *del-1* and induces *flp-18* and *mig-13* in VA12	[9] and this study
**VB**	Motor neuron	LIN-39 and MAB-5 regulate *del-1* in VB4-11	[9]
VC*	Motor neuron	LIN-39 promotes the survival of VC1-6	[18]
**VD**	Motor neuron	MAB-5 induces *ser-2* in VD12; EGL-5 represses *ser-2* in VD13	This study
**DD**	Motor neuron	EGL-5 represses *flp-13* in DD6	This study
**AVL**	Motor neuron	No regulation found	This study
**AQR**	Sensory	LIN-39 regulates *gcy-32*, *gcy-37*, and *F49H12*.*4* and the migration of its precursor	This study and [18]
**PQR**	Sensory	MAB-5 regulates the migration of PQR precursor	[20]
**AIY**	Interneuron	No regulation found	This study
**AVM**	Sensory	LIN-39 regulates *gcy-37* and the migration of AVM precursor	This study and [18]
**SDQ**	Interneuron	LIN-39 regulates *lad-2* and *flp-12* in SDQR and acts redundantly with MAB-5 to regulate them in SDQL; LIN-39 and MAB-5 regulate the migration of SDQR and SDQL precursors, respectively	This study and [18, 20]
**PDE**	Sensory	LIN-39 regulates *ser-2* and axonal growth	This study
**PVD**	Sensory	LIN-39 regulates *ser-2* and *F49H12*.*4*	This study
**HSN**	Motor neuron	EGL-5 regulates *tph-1* and *kal-1* and controls HSN migration and serotonin production	This study and [8]
PDA*	Motor neuron	EGL-5 regulates *cog-1* and *ace-3/4* and promotes Y-to-PDA transdifferentiation	[24]
**PDB**	Motor neuron	EGL-5 regulates *kal-1*	This study
DVB*	Motor neuron	EGL-5 regulates *flp-10*, *unc-47*, and *kal-1* and may promote K-to-DVB transdifferentiation	This study
**PHB**	Sensory	EGL-5 regulates *flp-4*; EGL-5 regulates *srb-6* in *ham-1(-)* mutants	This study and [25]
**LUA**	Interneuron	No regulation found	This study and [28]
PVC*	Interneuron	EGL-5 regulates *nmr-1* and *glr-1*	[6]
**PLM**	Sensory	EGL-5 regulates *mec-3*, which activates TRN fate genes	[7]
**DVA**	Interneuron	No regulation found	This study
**DVC**	Interneuron	No regulation found	This study
**PVR**	Interneuron	No regulation found	This study
PHC*	Sensory	PHP-3 and NOB-1 regulate *ida-1* and *dop-1*	This study
PLN*	Sensory	PHP-3 and NOB-1 regulate *lad-2* and *ser-2*	This study

Asterisks (*) indicate that Hox genes were known to regulate neurogenesis for these neurons or the possible involvement of Hox genes in their generation could not be ruled out.

From an evolutionary point of view, the importance of Hox genes in the development of the nervous system may have facilitated their strong conservation across species in animal lineages. On the other hand, the gain of additional Hox genes and paralogs in the genome (e.g., *C*. *elegans* has 6 Hox genes, *Drosophila* has 8, the amphioxus *Branchiostoma floridae* has 15, zebrafish has 32, and mice and humans have 39) may have contributed to the evolution of complexity in the nervous system.

### Hox genes promote neuronal fate specification through various mechanisms

Hox genes control neuronal fate specification by regulating terminal differentiation genes associated with the cell fate. In some neuron types (e.g., PVC, PVD, and PHC), Hox genes strongly regulate the expression of all terminal fate markers examined, whereas in other neuron types (e.g., PDE, PDB, and AVM), only a few fate markers are controlled by Hox genes. In some other cases (e.g., AVL, AIY, and LUA), we did not observe any regulation of fate markers by Hox genes. The different involvement of Hox genes in fate determination may be explained by the difference in expression levels. For example, *lin-39* expression is much stronger in AQR than in AIY, and *lin-39* regulates AQR but not AIY fate specification. Nevertheless, the role of a Hox gene in a neuron type is also likely affected by other factors (e.g., the presence of other cell fate regulators or extracellular signals).

Hox regulation of the terminal differentiation genes (or fate markers) could be either direct or indirect. For example, in the PLM neurons, EGL-5 promotes the specification of the touch receptor neuron (TRN) fate by facilitating the activation of the terminal selector *mec-3*, which in turn activates genes associated with the TRN fate [7]. In this study, we found that LIN-39 regulates the AQR and SDQ fate markers directly through the Hox-binding motifs identified in the regulatory regions of these genes. Similarly, Hox proteins control the subtype identities of cholinergic MNs by directly regulating the subtype-specific genes [9]. In the PVD neurons, however, LIN-39 indirectly controls the fate marker *ser-2* by regulating intermediate factors other than the terminal selector *mec-3*. Thus, Hox genes can function at different levels in the regulatory hierarchy of cell fate determination.

In addition to regulating genes associated with terminal neuronal fates, Hox proteins also control neuronal differentiation by inhibiting apoptosis (e.g., LIN-39 in VC neurons [18]), inducing transdifferentiation (e.g., EGL-5 in Y-to-PDA [24] and possibly K-to-DVB transdifferentiation), promoting axonal outgrowth (e.g., EGL-5 in PLM [6] and LIN-39 in PDE), and controlling cell migration (e.g., EGL-5 in HSN [8] and LIN-39 in the precursors of SDQR and AQR [18]) (Table 1). The versatile functions of the Hox genes suggest that they can function at multiple stages of neuronal differentiation.

### Hox genes are major regulators of subtype specification along the A-P axis

Neurons that share the same cell fate often differentiate into subtypes with specific morphological and functional features. Subtypes along the A-P axis often express different Hox genes, and these subtype-specific Hox genes have dual functions in both promoting general cell fate specification and inducing subtype features [6,7]. Our study revealed a few rules of Hox activities in subtype differentiation.

First, not all subtypes require Hox genes for fate specification. For example, although both PLM and AVM are TRN subtypes, only PLM requires Hox input for the differentiation of the TRN fate; EGL-5 in PLM but not LIN-39 in AVM promote the expression of TRN markers and the differentiation of the TRN fate [7]. Similarly, AQR requires LIN-39 for the specification of the oxygen sensory neuron fate, but PQR does not need MAB-5 for the same fate. The varying dependency on Hox activities for fate specification among the subtypes may be because different subtypes experience different regional cues (e.g., Wnt signals).

Second, certain Hox genes can trigger further differentiation of the subtype away from the general ground state of a neuronal fate. For example, our previous work in the TRNs found that the posterior Hox gene *egl-5* induces transcriptomic changes in the posterior subtype PLM, making it morphologically, molecularly, and functionally distinct from the anterior subtype ALM, which adopts a default TRN fate [6]. Similarly, in the ventral cord MNs (DA, VA, VD, and DD), *egl-5* alters the transcriptional program in the most posterior subtype, thus generating diversities among the MN types. EGL-5 does so through both repressing more anterior Hox genes (*lin-39* and *mab-5*) and direct regulation of the subtype-specific genes.

Third, in addition to the posterior dominance of subtype identities, we found evidence for middle body Hox genes generating molecular features in the middle body subtypes. For example, MAB-5 controls subtype-specific gene expression in the penultimate MN neurons along the A-P axis and represses *mig-13* expression in the DA and VA neurons located in the middle region of the body. LIN-39 induces *gcy-37* expression in the anteriorly located AVM neuron among the TRN subtypes. These findings extend the principles governing Hox regulation of subtype diversification beyond posterior dominance alone.

Moreover, during MN subtype differentiation, LIN-39, MAB-5, and EGL-5 could all act as both transcriptional activators and repressors depending on the target. This is likely because Hox proteins can be associated with both coactivators (e.g., CREB binding protein) and corepressors (e.g., histone deacetylases) as found in other organisms [34].

The function of Hox genes in regulating fate specification and diversifying neuronal subtypes along the A-P axis appeared to be highly conserved across species. In *Drosophila*, Hox genes *Ubx* and *abd-A* promote fate specification of ventral-abdominal (Va) neurons, while *abd-A* further induces subtype differentiation of Va neurons in the abdominal segments A2-4 [5]. In mouse spinal cord, *Hox5-Hox8* paralogous genes induce the specification of brachial lateral MNs [35]. Thus, deciphering the mechanisms by which Hox genes regulate terminal neuronal differentiation at the single cell level in the entire nervous system provides a firm ground for understanding the development of neuronal diversity.

## Materials and methods

### Strains and transgenes

*C*. *elegans* wild-type (N2) and mutant strains were maintained at 20°C as described previously [36]. Loss-of-function alleles used in this study include *lin-39(n1760)*, *mab-5(gk670)*, *egl-5(u202)*, *php-3(ok919)*, *nob-1(ct230)*, *ceh-20(u843)*, and *unc-30(e191)*. Among them, *ceh-20(u843)* we previously isolated carries the Q250Stop mutation and is a strong *lf* allele [37]. For the analysis of double mutants, we crossed the transgenes into the MT7236 *lin-39(n1760) egl-5(n945)*, CB4711 *mab-5(e1239) egl-5(n945)*, and MT7419 *lin-39(n1760) mab-5(e1239)/sma-3(e491) mab-5(e1239)* strains. Transgenes used in this study were all described in Results. Some strains were obtained from the Caenorhabditis Genetics Center.

To create the endogenous GPF knock-in *ceh-13*::*GFP(unk27)* and *php-3*::*GFP(unk25)* strains, we adopted a published CRISPR/Cas9 gene editing protocol [38]. For *ceh-13*, we used 5’-TCACCGACATCGTCATGCAG-3’ as the Cas9 target sequence and GFP-coding sequences amplified from pPD95.75 and attached with 250-bp of flanking homologous arm sequences, which are designed to insert the GFP before the stop codon. To avoid the cutting of the repair template, we introduced synonymous mutations in the target region in the repair template. Recombinant spCas9 (NEB) and single guide RNA (made using the sgRNA synthesis kit from NEB) together with the repair template (as PCR products) were injected into the wild-type animals. Successfully edited progeny were identified by worm PCR. Similarly, for *php-3* we used 5’-CCTGGATTGGATTAACTCGA-3’ as the target sequence. Since the GFP sequence insertion breaks the target sequence in the repair template, we did not introduce any synonymous mutations to the repair template.

To cross into animals carrying the *lin-39(n1760)* null allele, which caused the vulvaless phenotype, we created TU6438 *egl-5(u202); lin-39(n1760); unkEx1[lin-39(+); ceh-22p*::*GFP]* and CGZ1 *lin-39(n1760); unkEx1[lin-39(+); ceh-22p*::*GFP]* strains by first injecting the fosmid WRM0630bD02 that contains the wild-type *lin-39* locus into the MT7236 *lin-39(n1760) egl-5(n945)* strain to restore vulval development in TU6438 and then mating them with *lin-39(n1760)* males to obtain CGZ1. After crossing various transgenes into TU6438 and CGZ1, we picked the animals that lost the extrachromosomal array *unkEx1* and obtained homozygous Hox mutants with the transgene.

To map the expression of Hox genes, we crossed the GFP reporters for Hox genes, including *wgIs18[lin-39*::*EGFP]*, *wgIs27[mab-5*::*EGFP]*, *wgIs54[egl-5*::*EGFP]*, *php-3*::*GFP(unk25)*, and *stIs10286[nob-1*::*GFP]* with mCherry reporter strains for the neurotransmitter identities, including *otIs518[eat-4(fosmid)*::*SL2*::*mCherry*::*H2B]*, *otIs544[cho-1(fosmid)*::*SL2*::*mCherry*::*H2B]*, and *otIs564[unc-47(fosmid)*::*SL2*::*H2B*::*mChopti]* for glutamatergic, cholinergic, and GABAergic neurons, respectively [39–41]. We also crossed the Hox reporters with *otIs181[dat-1p*::*mCherry]* and *vsIs97[tph-1p*::*DsRed2]* for the identification of dopaminergic and serotonergic neurons [42,43], respectively. Hox-expressing neurons were identified based on the position of the GFP-expressing nuclei and the overlapping with specific neurotransmitter identity reporter according to the neurotransmitter map developed by the Hobert lab (https://www.hobertlab.org/neurotransmitter-map/). We only counted the neurons that can be consistently and unambiguously identified in over ten animals. Weak expression that cannot be reliably observed or neurons with ambiguous identities were not included in our results.

### Promoter bashing and *cis*-regulatory element analysis

Promoters upstream of the start codon of the cell fate marker genes were cloned from the N2 genomic DNA and inserted into pPD95.75 vector through Gibson Assembly (New England Biolabs, Ipswich, MA, U.S.). To delete specific *cis*-regulatory elements in the promoter, we used the Q5 site-directed mutagenesis kit (NEB) to generate constructs that lacked the regulatory sites. All promoter-GFP constructs and their variants were injected into the wild-type animals together with a co-injection marker, pCFJ104 [*myo-3p*::*mCherry*]. The resulted stable lines were analyzed for GFP expression patterns at day 1 adult stage. At least two independent lines were obtained for each construct, and at least 40 animals were examined for each line. The percentage of animals showing GFP expression were shown as the mean ± SD of different lines.

### Fluorescent imaging, phenotype scoring, and statistical analysis

Fluorescent imaging was done on a Leica DMi8 inverted microscope equipped with a Leica DFC7000 GT or a Leica K5 monochrome camera. To analyze gene expression patterns, we imaged at least 30 day-one adult animals for each strain and calculated the percentages of animals showing the expression of the reporter. Three biological replicates were carried out for each imaging experiment, and *Chi*-square test were used to determine whether there is a significant difference between the wild-type and the mutants (double asterisks for *p* < 0.01 in Figures). Raw data can be found in S6 Table. Representative images were shown. For A/PQR and SDQL/R mispositioning in the Hox mutants, we scored the neuron as “mispositioned” when we found that the fluorescent reporter labeled the correct number of neurons as in the wild type but one of the neurons had a posteriorly or anteriorly displaced cell body in *lin-39(-)* or *mab-5(-)* mutants, respectively.

## Supporting information

S1 Table Comparison of Hox expression patterns from different studies. Neurons found to express Hox genes in the scRNA-seq data at L4 stage from Taylor *et al*., 2021 [1], in the Reilly *et al*., 2020 study [2], and in this study were compared. For scRNA-seq data, expression values were extracted from Wormbase WS282. Red indicates the expression that are consistent between our results and other studies. Blue indicates the expression found in our study that show discrepancy with other studies.
(XLSX)

S2 Table Hox expression in embryonic neurons found by the scRNA-seq of the embryos. Expression values were extracted from https://cello.shinyapps.io/celegans/ based on the scRNA-seq data from Packer *et al*., 2019 [1]. Only the neurons with adjusted.tpm.estimate > 0 were shown. Red indicates the expression were confirmed by our analysis using the fluorescent reporters.
(XLSX)

S3 Table Hox expression in embryonic lineages found by the scRNA-seq of the embryos. Expression values were extracted from https://cello.shinyapps.io/celegans/ based on the scRNA-seq data from Packer *et al*., 2019 [1]. Only the AB lineage decedents with adjusted.tpm.estimate > 0 were shown.
(XLSX)

S4 Table LIN-39 and MAB-5 did not regulate the fate specification of AIY and AVL neurons, respectively. Penetrance of GFP expression from the corresponding reporters in *lin-39(n1760)* and *mab-5(gk670)* mutants.
(XLSX)

S5 Table Summary of Hox functions in neuronal differentiation in *C*. *elegans*. The content of this table is the same as Table 1, except that the regulatory events are organized by individual Hox genes for easy reference.
(XLSX)

S6 Table Raw scoring data for expression patterns shown in Figs 2–5. At least 30 day 1 adult animals are used in each test.
(XLSX)

S1 Fig The expression of *ceh-13* in the embryos but not larva. (A) The expression of the fosmid-based reporter *wgIs756[ceh-13*::*EGFP]* in early embryos and the lack of any expression in larva. (B) The expression of endogenous *ceh-13*::*GFP* knock-in, which was generated by CRISPR/Cas9-mediated gene editing, in the embryos at the bean stage and the comma stage. The expression faded away in the 3-fold stage embryos and was not observed in larva. (C) Super weak and variable expression of *ceh-13*::*GFP* in the ventral nerve cord motor neurons (arrows) in adults. Scale bars = 20 μm.
(TIF)

S2 Fig The expression of *lin-39* in the ventral cord motor neurons (MNs). (A) The expression of *lin-39* reporter in GABAergic MNs, including VD3-12 and DD2-6. The nuclei of GABAergic MNs were labeled by neurotransmitter identity marker *unc-47*. (B) The expression of *lin-39* in cholinergic MNs, including DA2-5, DB2-7, VA3-8, VB4-9, AS2-8, VC1-3, and VC6. *lin-39* was also expressed in VC4 and VC5, which are not cholinergic. Scale bars = 20 μm.
(TIF)

S3 Fig The expression of *mab-5* in the ventral cord MNs. (A) The expression of *mab-5* reporter in GABAergic MNs, including VD2-12 and DD2-6. (B) The expression of *mab-5* in cholinergic MNs, including DA4-8, DB5-7, VA6-11, VB8-11, AS5-11, VC3, and VC6. *mab-5* was also expressed in VC4 and VC5, which are not cholinergic. Scale bars = 20 μm.
(TIF)

S4 Fig Schematic representation of *nob-1* and *php-3* loci and the structure of their reporters. Black boxes represent exons and black lines for introns. Brown boxes represent the untranslated regions (UTRs). *stIs10286[nob-1*::*GFP]* is a translational reporter with *unc-54* 3’UTR. *php-3*::*GFP(unk25)* is a GFP knock-in at the endogenous locus of *php-3*.
(TIF)

S5 Fig LIN-39 does not regulate TRN fate markers in AVM. (A) AVM was posteriorly displaced in *lin-39(n1760)* mutants, but the expression of TRN fate marker *uIs115[mec-17p*::*TagRFP]* was not affected in *lin-39* mutants. (B) The displaced AVM also expressed the TRN fate marker *zdIs5[mec-4p*::*GFP]* in *lin-39* mutants. Scale Bars = 100 μm.
(TIF)

S6 Fig The loss of PVD and PDE fate marker expression in *lin-39* mutants. (A) The penetrance for the expression of the PVD and PDE fate marker *otIs138[ser-2p3*::*GFP]*, the PVD fate marker *wdIs51[F49H12*.*4p*::*GFP]*, and dopaminergic identity marker *egIs1[dat-1p*::*GFP]* in *lin-39(n1760)*, *mab-5(gk670)*, and *ceh-20(u843)* mutants. (B) The expression of the PDEsh markers *unkEx83[C34B2*.*9p*::*GFP]* in *lin-39* and *ceh-20* mutants.
(TIF)

S7 Fig LIN-39 motif in the promoters of neuronal fate markers. (A) The sequences of functional Hox sites identified in this study (Fig 3), which likely mediate the regulation of neuronal fate markers by LIN-39. (B) The sequence logo of LIN-39 binding sites generated from ChIP-seq data. These logos were downloaded from http://cisbp.ccbr.utoronto.ca/.
(TIF)

S8 Fig ChIP-seq signal for LIN-39 and EGL-5 in the promoter regions of neuronal fate markers. ChIP-seq data from the modENCODE project were retrieved from Wormbase (WS283) and visualized using the Jbrowse at the promoter regions of various neuronal fate markers. The signals from the “LIN-39 Combined (GFP ChIP) recalled peaks” (A) and “EGL-5 Combined (GFP ChIP) recalled peaks” (B) tracks were shown. In (A), arrows indicate the peaks that cover the region where the functional LIN-39 sites were found.
(TIF)

S9 Fig DVA, DVC, and PVR fates are not affected in *php-3* and *nob-1* mutants. (A) The expression of DVA fate markers, *dbl-1* and *flp-11*, were not affected in *php-3(ok919)* and *nob-1(ct230)* mutants. The numbers in the parentheses indicate the number of cells examined; 100% indicate all of them showed normal expression. For *nob-1(ct230)* mutants, animals with severe malformation of the tail were excluded. (B) The expression of DVC fate marker *srab-12* and PVR fate marker *flp-10* were not affected in *php-3* mutants. Scale Bars = 20 μm.
(TIF)

S10 Fig Hox regulation of MN subtype marker expression in the middle body region. (A) Derepression of *mig-13* in the cholinergic MN subtypes in the middle body region, including DA5-8 and VA7-11 in *mab-5(gk670)* mutants. Asterisk indicates the position of the vulva. Scale Bars = 20 μm. (B) Variable loss of *unc-129* expression in a few anterior DA and DB neurons, including DB2 and DB4, in *lin-39(n1760)* mutants. Dashed circles indicate the loss of GFP expression in corresponding neurons. Scale Bars = 100 μm. (C) Variable loss of *del-1* expression in a few anterior VA and VB neurons, including VA4-5 and VB5-6, in *lin-39* mutants. (D) Variable loss of *ser-2* expression in very few VD neurons, including VD8, in *lin-39(n1760)* and *unc-30(e191)* mutants. Scale Bars = 100 μm.
(TIF)
