# Supplementary figures and images for: Nervous system-wide analysis of Hox regulation of terminal neuronal fate specification in Caenorhabditis elegans
Source: PLoS Genet. 2022 Feb 28;18(2):e1010092. doi: 10.1371/journal.pgen.1010092 (PMC8912897; doi:10.1371/journal.pgen.1010092)

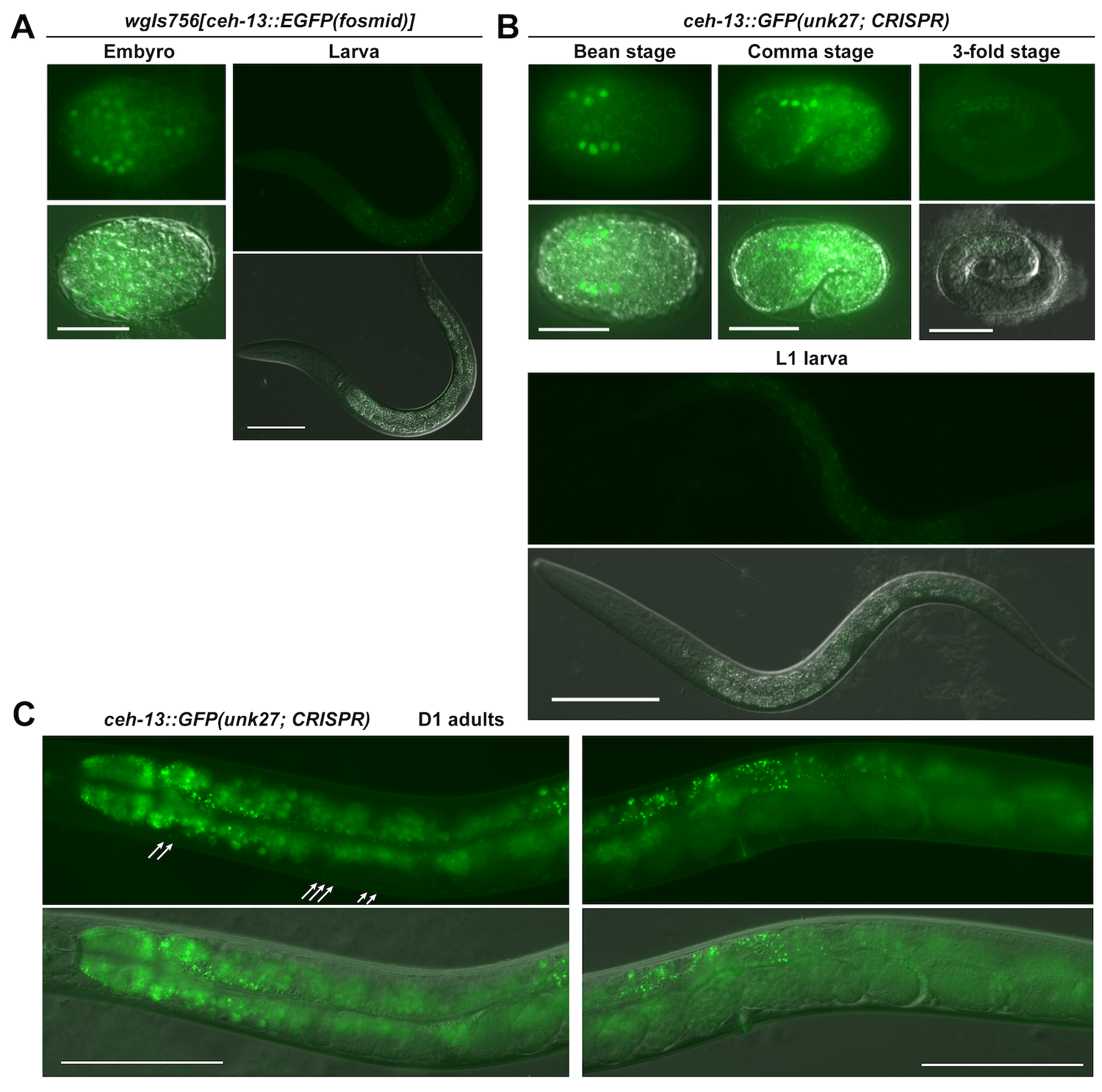

Supplement: S1 Fig — (A) The expression of the fosmid-based reporter wgIs756[ceh-13::EGFP] in early embryos and the lack of any expression in larva. (B) The expression of endogenous ceh-13::GFP knock-in, which was generated by CRISPR/Cas9-mediated gene editing, in the embryos at the bean stage and the comma stage. The expression faded away in the 3-fold stage embryos and was not observed in larva. (C) Super weak and variable expression of ceh-13::GFP in the ventral nerve cord motor neurons (arrows) in adults. Scale bars = 20 μm. (TIF) [file pgen.1010092.s007.tif]

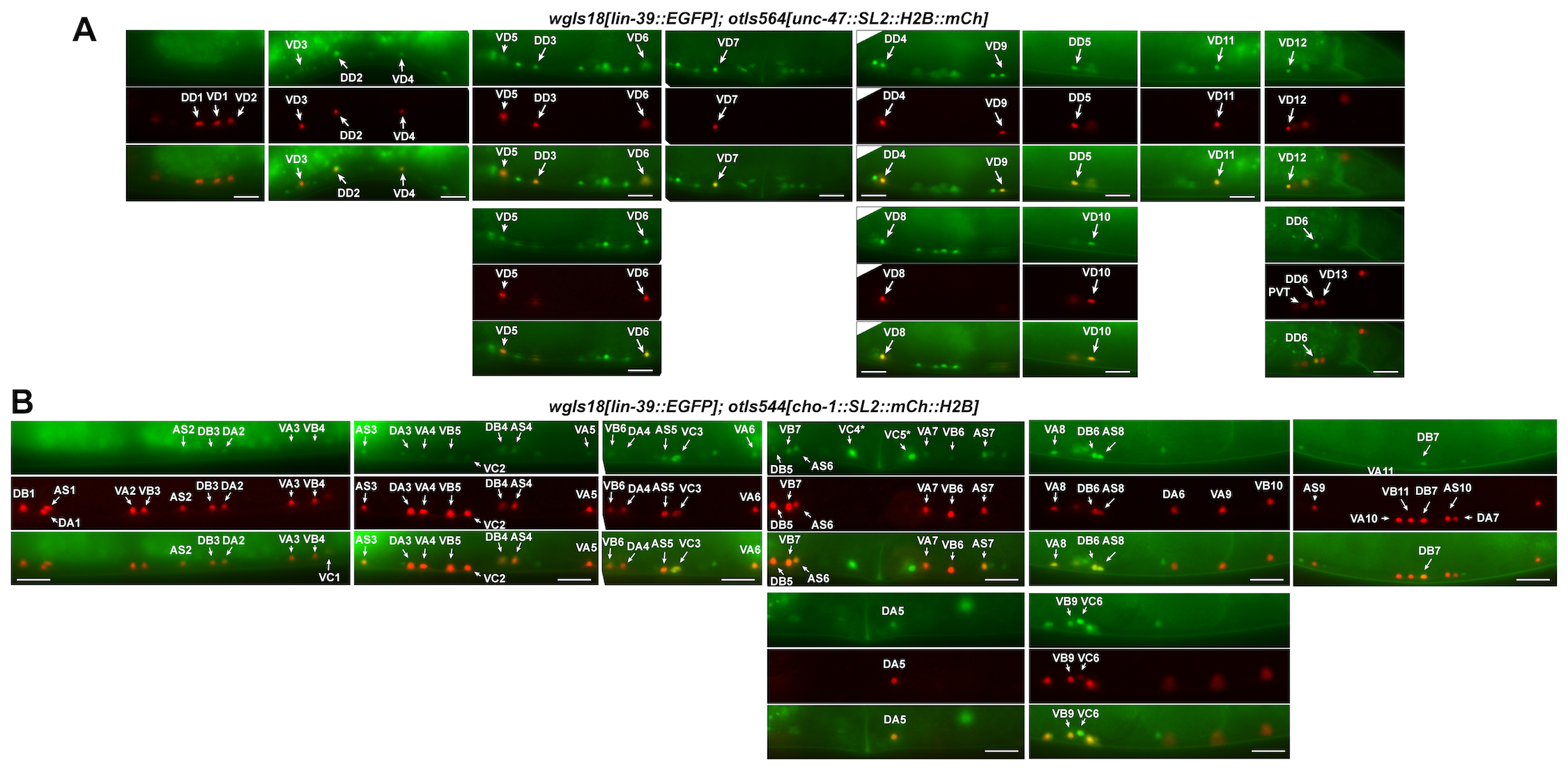

Supplement: S2 Fig — (A) The expression of lin-39 reporter in GABAergic MNs, including VD3-12 and DD2-6. The nuclei of GABAergic MNs were labeled by neurotransmitter identity marker unc-47. (B) The expression of lin-39 in cholinergic MNs, including DA2-5, DB2-7, VA3-8, VB4-9, AS2-8, VC1-3, and VC6. lin-39 was also expressed in VC4 and VC5, which are not cholinergic. Scale bars = 20 μm. (TIF) [file pgen.1010092.s008.tif]

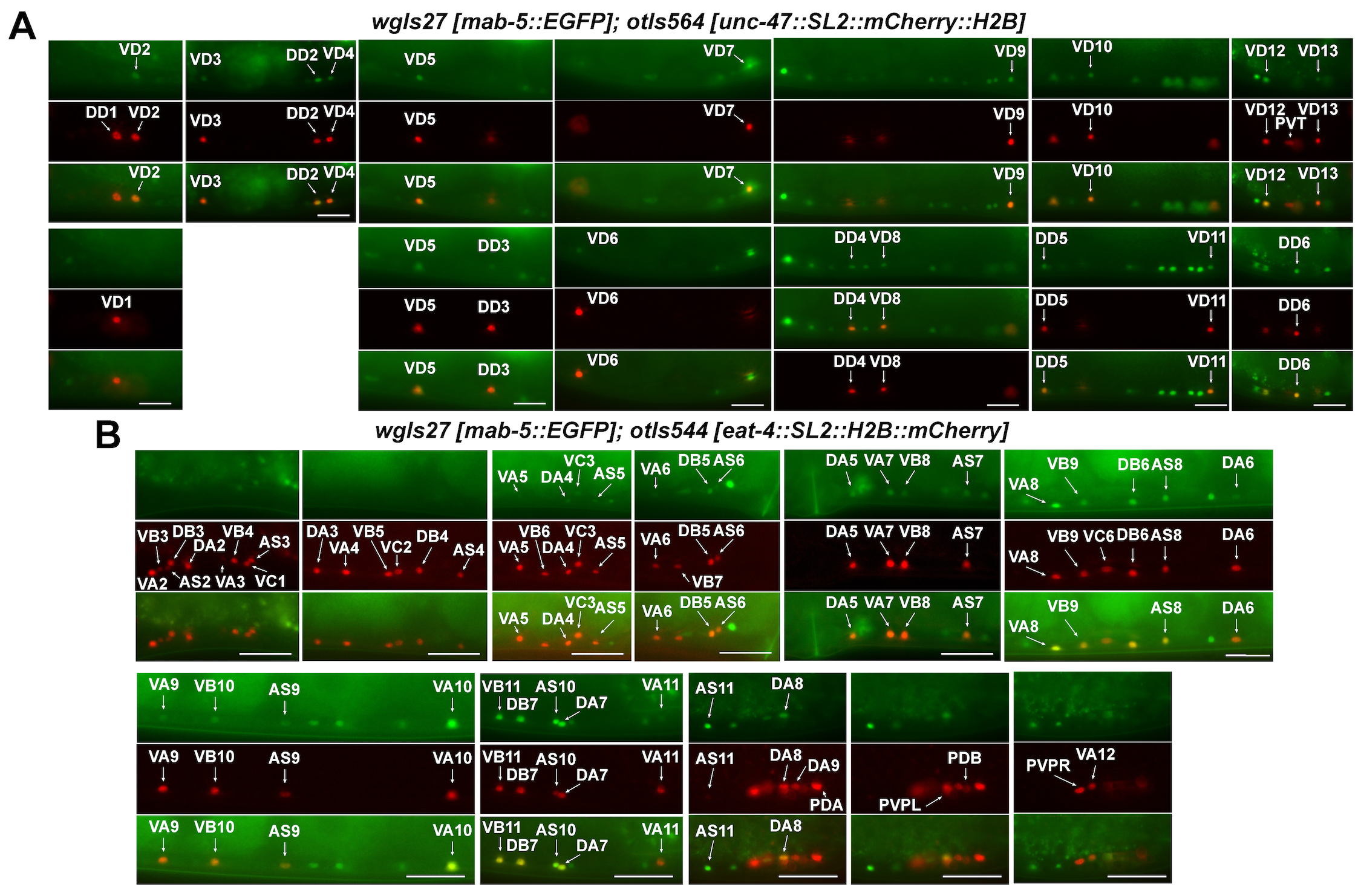

Supplement: S3 Fig — (A) The expression of mab-5 reporter in GABAergic MNs, including VD2-12 and DD2-6. (B) The expression of mab-5 in cholinergic MNs, including DA4-8, DB5-7, VA6-11, VB8-11, AS5-11, VC3, and VC6. mab-5 was also expressed in VC4 and VC5, which are not cholinergic. Scale bars = 20 μm. (TIF) [file pgen.1010092.s009.tif]

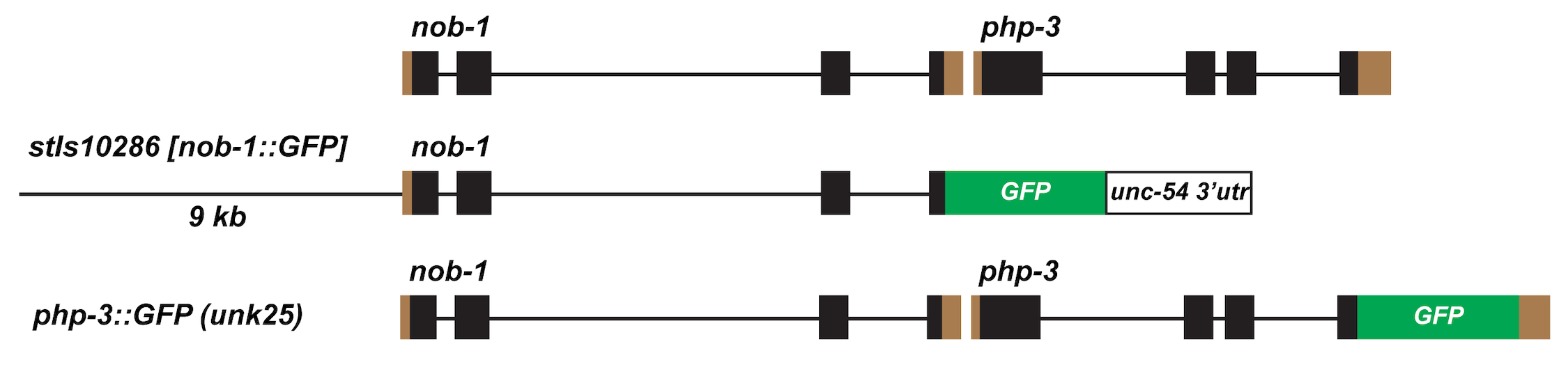

Supplement: S4 Fig — Black boxes represent exons and black lines for introns. Brown boxes represent the untranslated regions (UTRs). stIs10286[nob-1::GFP] is a translational reporter with unc-54 3’UTR. php-3::GFP(unk25) is a GFP knock-in at the endogenous locus of php-3. (TIF) [file pgen.1010092.s010.tif]

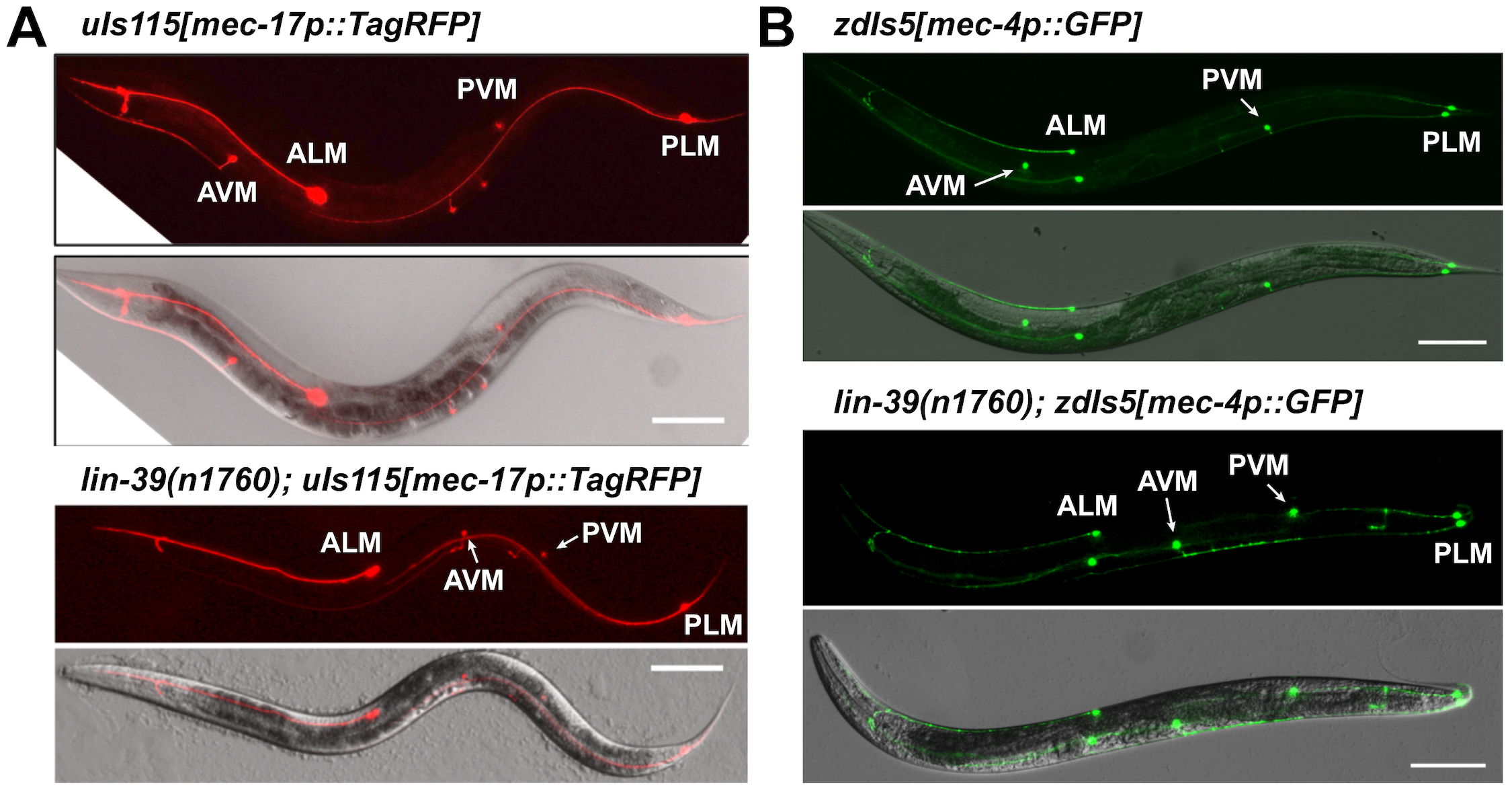

Supplement: S5 Fig — (A) AVM was posteriorly displaced in lin-39(n1760) mutants, but the expression of TRN fate marker uIs115[mec-17p::TagRFP] was not affected in lin-39 mutants. (B) The displaced AVM also expressed the TRN fate marker zdIs5[mec-4p::GFP] in lin-39 mutants. Scale Bars = 100 μm. (TIF) [file pgen.1010092.s011.tif]

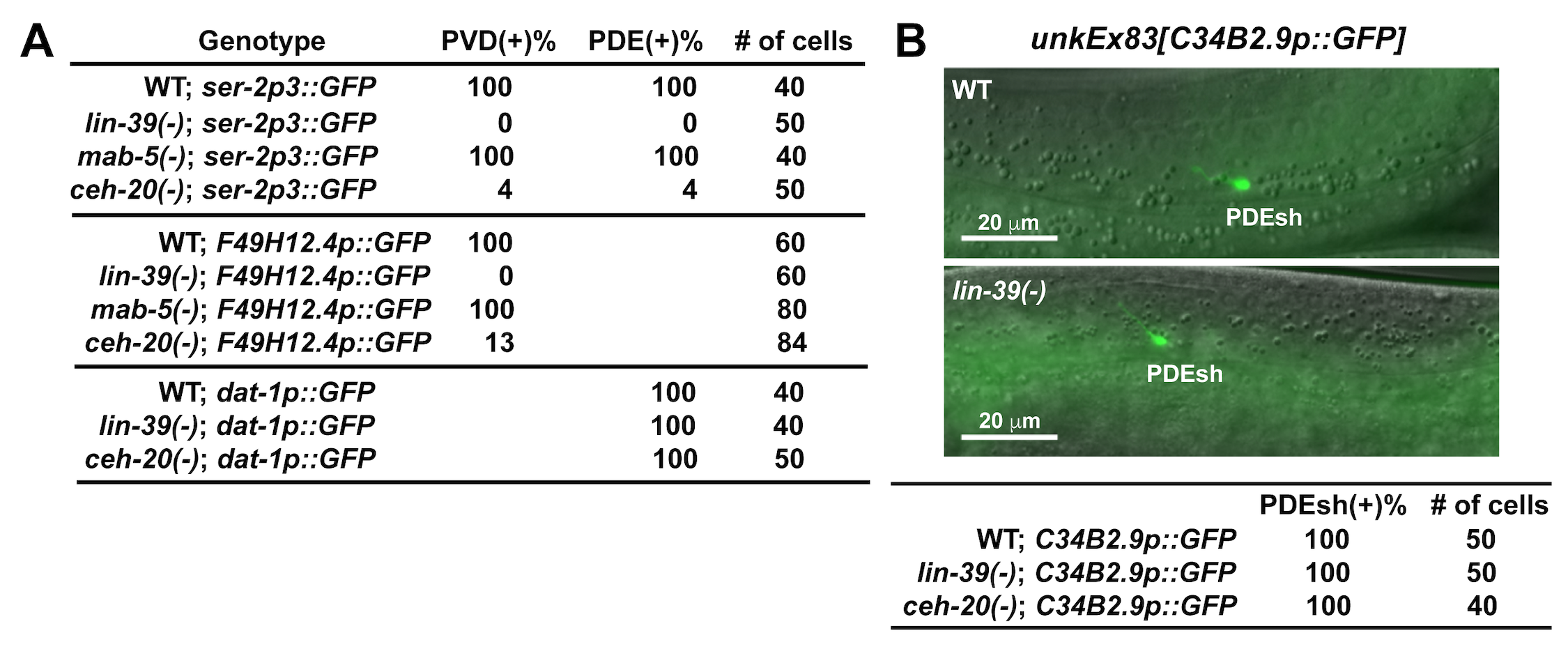

Supplement: S6 Fig — (A) The penetrance for the expression of the PVD and PDE fate marker otIs138[ser-2p3::GFP], the PVD fate marker wdIs51[F49H12.4p::GFP], and dopaminergic identity marker egIs1[dat-1p::GFP] in lin-39(n1760), mab-5(gk670), and ceh-20(u843) mutants. (B) The expression of the PDEsh markers unkEx83[C34B2.9p::GFP] in lin-39 and ceh-20 mutants. (TIF) [file pgen.1010092.s012.tif]

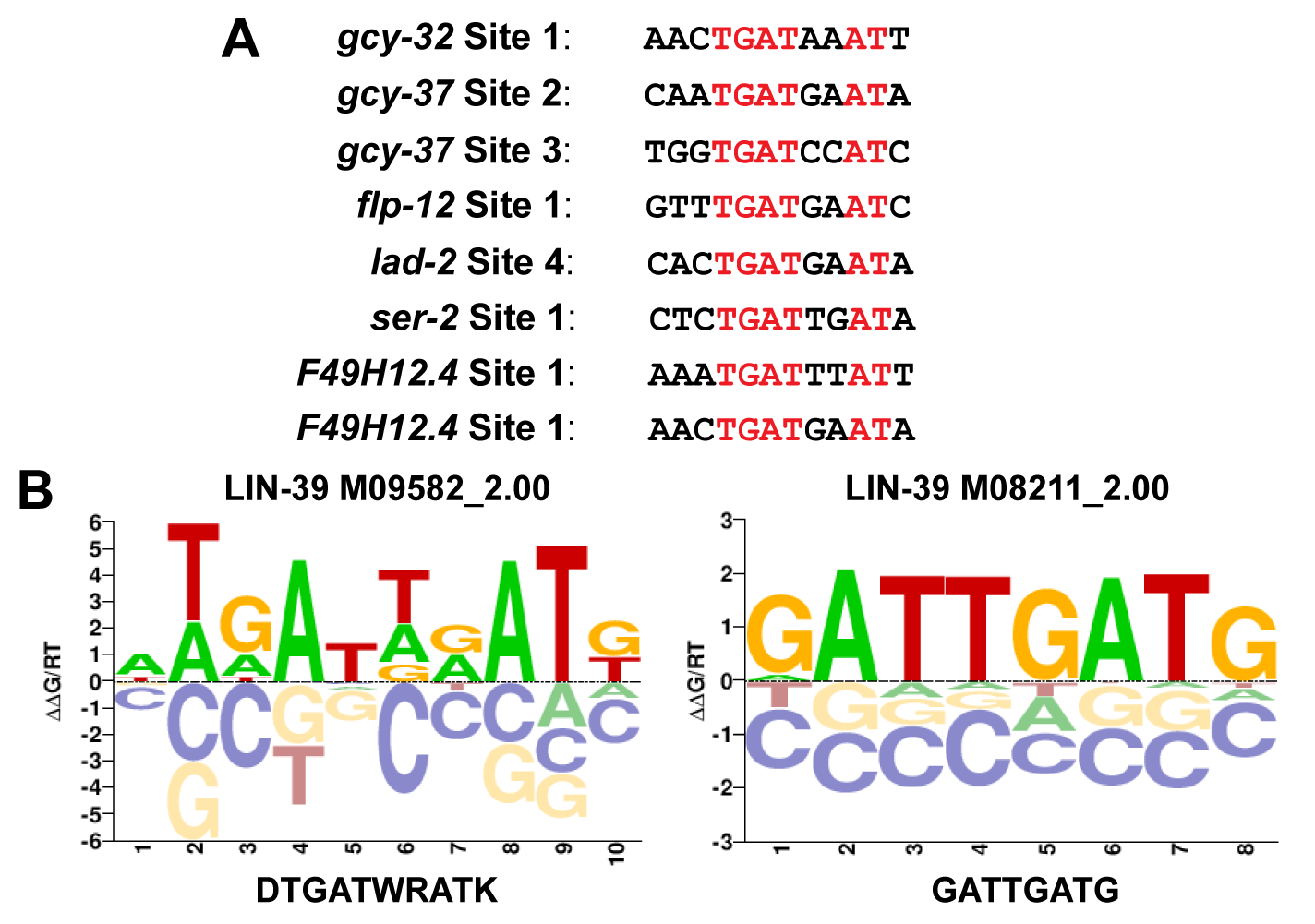

Supplement: S7 Fig — (A) The sequences of functional Hox sites identified in this study (Fig 3), which likely mediate the regulation of neuronal fate markers by LIN-39. (B) The sequence logo of LIN-39 binding sites generated from ChIP-seq data. These logos were downloaded from http://cisbp.ccbr.utoronto.ca/. (TIF) [file pgen.1010092.s013.tif]

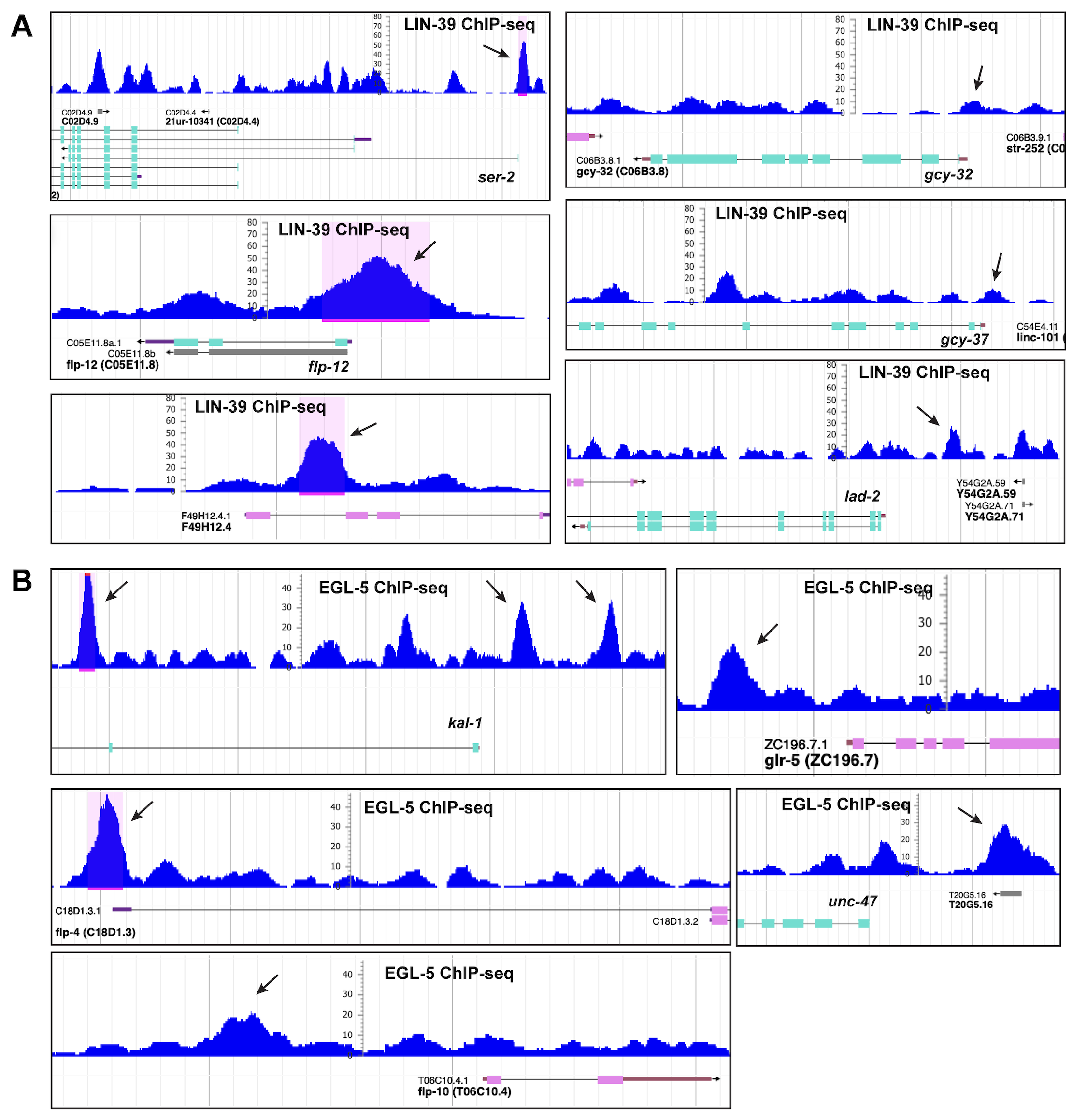

Supplement: S8 Fig — ChIP-seq data from the modENCODE project were retrieved from Wormbase (WS283) and visualized using the Jbrowse at the promoter regions of various neuronal fate markers. The signals from the “LIN-39 Combined (GFP ChIP) recalled peaks” (A) and “EGL-5 Combined (GFP ChIP) recalled peaks” (B) tracks were shown. In (A), arrows indicate the peaks that cover the region where the functional LIN-39 sites were found. (TIF) [file pgen.1010092.s014.tif]

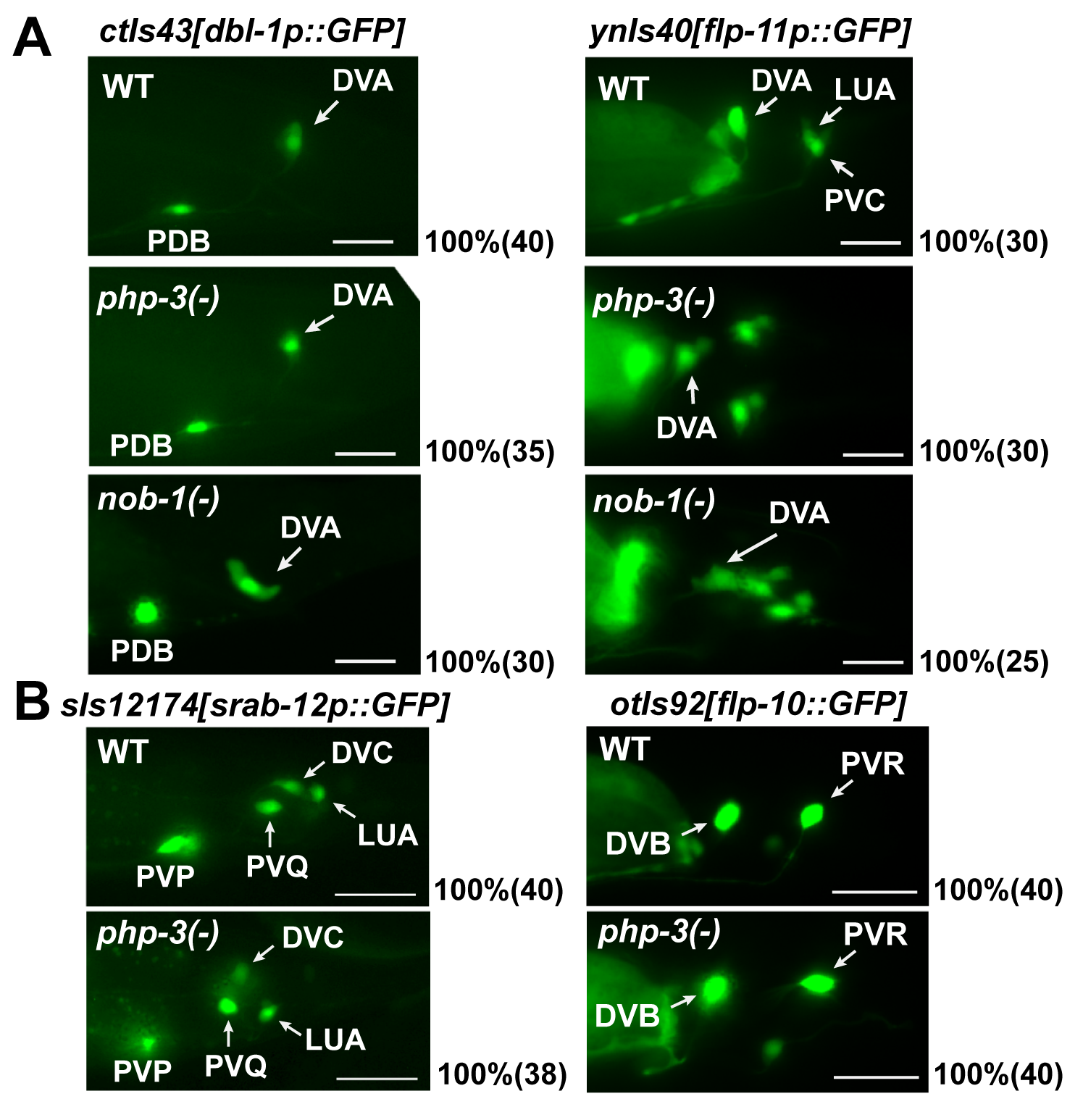

Supplement: S9 Fig — (A) The expression of DVA fate markers, dbl-1 and flp-11, were not affected in php-3(ok919) and nob-1(ct230) mutants. The numbers in the parentheses indicate the number of cells examined; 100% indicate all of them showed normal expression. For nob-1(ct230) mutants, animals with severe malformation of the tail were excluded. (B) The expression of DVC fate marker srab-12 and PVR fate marker flp-10 were not affected in php-3 mutants. Scale Bars = 20 μm. (TIF) [file pgen.1010092.s015.tif]

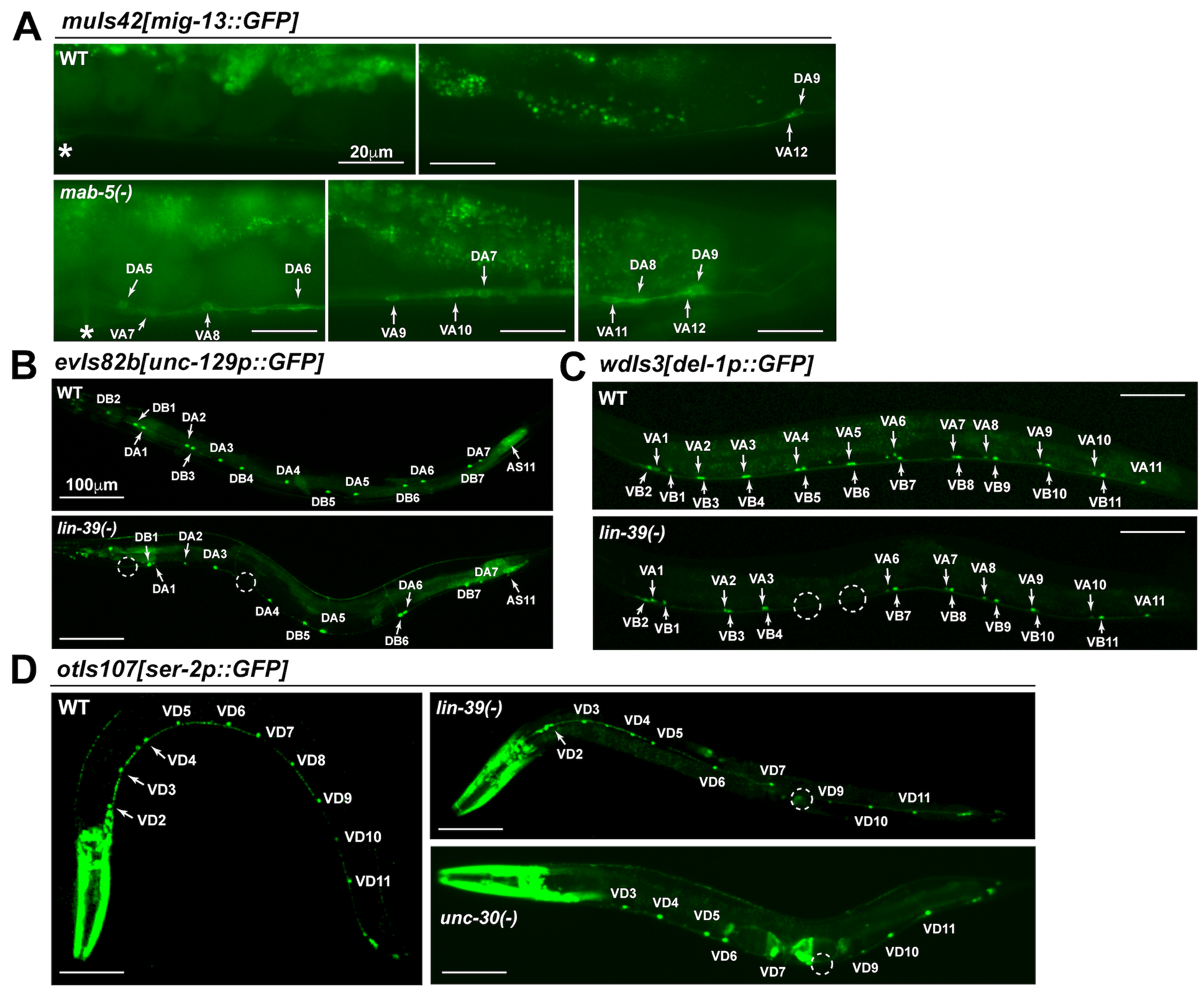

Supplement: S10 Fig — (A) Derepression of mig-13 in the cholinergic MN subtypes in the middle body region, including DA5-8 and VA7-11 in mab-5(gk670) mutants. Asterisk indicates the position of the vulva. Scale Bars = 20 μm. (B) Variable loss of unc-129 expression in a few anterior DA and DB neurons, including DB2 and DB4, in lin-39(n1760) mutants. Dashed circles indicate the loss of GFP expression in corresponding neurons. Scale Bars = 100 μm. (C) Variable loss of del-1 expression in a few anterior VA and VB neurons, including VA4-5 and VB5-6, in lin-39 mutants. (D) Variable loss of ser-2 expression in very few VD neurons, including VD8, in lin-39(n1760) and unc-30(e191) mutants. Scale Bars = 100 μm. (TIF) [file pgen.1010092.s016.tif]
